# Functional Characterization of Transcription Factor Motifs Using Cross-species Comparison across Large Evolutionary Distances

**DOI:** 10.1371/journal.pcbi.1000652

**Published:** 2010-01-29

**Authors:** Jaebum Kim, Ryan Cunningham, Brian James, Stefan Wyder, Joshua D. Gibson, Oliver Niehuis, Evgeny M. Zdobnov, Hugh M. Robertson, Gene E. Robinson, John H. Werren, Saurabh Sinha

**Affiliations:** 1 Department of Computer Science, University of Illinois at Urbana-Champaign, Urbana, Illinois, United States of America; 2 Department of Genetic Medicine and Development, University of Geneva Medical School, and Swiss Institute of Bioinformatics, Geneva, Switzerland; 3 School of Life Sciences, Arizona State University, Tempe, Arizona, United States of America; 4 Department of Biology, University of Osnabrück, Osnabrück, Germany; 5 Department of Entomology, University of Illinois at Urbana-Champaign, Urbana, Illinois, United States of America; 6 Institute for Genomic Biology, University of Illinois at Urbana-Champaign, Urbana, Illinois, United States of America; 7 Department of Biology, University of Rochester, Rochester, New York, United States of America; University of British Columbia, Canada

## Abstract

We address the problem of finding statistically significant associations between *cis*-regulatory motifs and functional gene sets, in order to understand the biological roles of transcription factors. We develop a computational framework for this task, whose features include a new statistical score for motif scanning, the use of different scores for predicting targets of different motifs, and new ways to deal with redundancies among significant motif–function associations. This framework is applied to the recently sequenced genome of the jewel wasp, *Nasonia vitripennis*, making use of the existing knowledge of motifs and gene annotations in another insect genome, that of the fruitfly. The framework uses cross-species comparison to improve the specificity of its predictions, and does so without relying upon non-coding sequence alignment. It is therefore well suited for comparative genomics across large evolutionary divergences, where existing alignment-based methods are not applicable. We also apply the framework to find motifs associated with socially regulated gene sets in the honeybee, *Apis mellifera*, using comparisons with *Nasonia*, a solitary species, to identify honeybee-specific associations.

## Author Summary

We develop a computational pipeline for predicting the functions of transcription factor motifs, through DNA sequence analysis. The pipeline is applied to the newly sequenced genome of the jewel wasp, *Nasonia vitripennis*. It exploits the wealth of molecular data available in another insect species, the fruitfly *Drosophila melanogaster*, and uses cross-species comparison to its advantage. Our main contribution is to show how this can be done despite the large evolutionary divergence between the two species. The methodology presented here may be applied more generally to other scenarios (genomes) where comparative regulatory genomics must deal with large evolutionary divergences.

## Introduction

Computational discovery and analysis of gene regulatory networks begins with the characterization of transcription factor (TF) motifs, through experimental or computational means. The next task of characterizing the biological functions regulated by these motifs is crucial for gaining broad, systems-level insights about the regulatory network, and has been the subject of several studies in recent years [1]–[3]. We present a general framework for discovering such motif – function associations through genome sequence analysis, while using evolutionary conservation as a guide. Evolutionary comparisons in this framework are carried out without relying upon alignment of non-coding sequences, making the framework especially well suited for species that are greatly diverged from their nearest sequenced relatives.

Starting with a list of TF motifs, a researcher is often faced with the task of annotating putative binding sites matching those motifs, the so-called “motif scanning” [4] task. The predicted binding sites may then be used to annotate a set of genes (typically genes that are proximal to the sites) as being putative regulatory targets of the motif. Such a set of (predicted) target genes of a TF is called its “motif module” [5]. A motif module is thus a part of the gene regulatory network, representing the direct regulatory targets of a TF. Prediction of motif modules has been the focus of numerous studies in the past [6]–[8]. In a later section (“Motif scanning methods”), we briefly review existing approaches to this problem, most of which are based on finding sites whose quality of match to the motif exceeds a threshold, or locations where clusters of above-threshold matches are found. Each of these approaches has its merits and problems, and it is not clear which method ought to be used in practice. We examine this issue systematically, while proposing a new statistical score for motif scanning, and find different methods to be most efficacious for predicting the motif module for different TFs.

A motif module may be tested for statistical enrichment for any given gene set, such as genes in a Gene Ontology (GO) functional category [9], a metabolic or signal transduction pathway [10], or genes coordinately expressed in a particular condition [11]–[12]. Such statistical enrichment can shed light on possible biological roles of the motif. A compendium of statistical associations between motifs and functions is called a “motif function map” [2]. This map is a potential starting point for researchers exploring the *cis*-regulatory basis of a particular biological process [5]. It may be constructed by straight-forward statistical procedures for significance of overlap between a motif module and a functionally coherent gene set [1]–[2]. One problem faced by such a construction is that of redundant associations, e.g., where two or more functional sets are minor variants of each other. We present a new statistical approach to deal with this problem, which examines the significance of a motif – function association conditional on another association.

Prior studies have helped lay the informatics foundations of motif module and motif function map prediction in genomes with the greatest wealth of molecular data, such as yeast, fruitfly, mouse and human. Factors facilitating their success have included the availability of experimentally characterized motifs [13]–[14], gene function annotations [9], the opportunity to use alignment-based comparison among closely related species [15] and other sources of information such as chromatin immunoprecipitation-based binding data [16], tissue-specific gene expression data [1],[17], etc. However, to a researcher interested in gene regulatory networks of a less studied genome, that lacks the wealth of molecular data listed above, the previously published frameworks for motif analysis are not directly applicable. A special framework is needed for motif function map construction in such genomes, that can exploit useful prior information, such as motifs, genome sequence and gene function annotation, from a *distantly* related species. One such framework is developed and presented here.

An important lesson from recent work on genome-wide *cis*-regulatory analysis has been the critical role of comparative genomics [17]–[18] in curbing false positive predictions. Cross-species comparison may be used directly in motif scanning, by highlighting putative binding sites whose conservation is revealed by alignments [19]. It is also worthwhile to compare motif modules across different species, in the hope that evolutionarily conserved components of a module will represent more reliable motif – target relationships [20]. Yet another plausible way to exploit comparative genomics, and one that we explore here, is to compare motif function maps across species. Here, the motif module and motif function predictions are done separately in each species, and motif – function associations that are evolutionarily conserved are highlighted. This approach may have the advantage of detecting true motif – function relationships even if the underlying motif module is not found to be sufficiently well conserved evolutionarily, perhaps due to errors in the its computational prediction. This is the novel comparative genomics paradigm proposed and implemented in our framework, which we use to achieve more specific predictions, without relying on non-coding sequence alignment or the availability of genome sequences of closely related species.

We illustrate the use of our new framework by predicting motif functions in the recently sequenced genome of the jewel wasp, *Nasonia vitripennis* (Insecta: Hymenoptera), the first of a parasitoid species to be sequenced [21]. Even though the evolutionary divergence of *Nasonia* from its closest sequenced relative, the honeybee *Apis mellifera* (∼180 Myrs, [21]), and from the fruitfly, *Drosophila melanogaster* (∼300 Myrs, [21]), precludes alignment-based comparison of non-coding sequences, we are able to exploit these two genomes as well as the vast knowledge base in *Drosophila* to make reliable predictions in *Nasonia*.

## Results

### Overview

We begin with an outline of the major contributions of this work, pointing out the specific challenges that needed to be addressed.

#### Computational pipeline development and evaluation

There are two major components here.

1. Motif function prediction in single species: *First*, we examine the motif scanning problem, i.e., predicting regulatory targets of a TF, given its binding specificity (motif). We propose a new statistical score, based on hidden Markov models, for this problem. We implement this score, as well as two alternative scores that capture the gist of existing statistical approaches to the problem [7], [22]–[24]. *Second*, the highest scoring target genes of a motif are tested for association with specific functions, i.e., Gene Ontology (GO) [9] categories. Since this step in its conventional form tends to report motif associations with numerous mutually redundant GO categories [2], we propose a new statistical approach, based on an extension of the Hypergeometric test, to trim the list of significant associations to a non-redundant list. *Finally*, we apply the above two steps on a well studied genome (e.g., *Drosophila melanogaster*) where motifs have been discovered and genes have been annotated with GO categories, to choose one of the three motif scanning scores as the most appropriate one for each motif. The selected motif scanning score, along with tools for the first two steps above, can now be used to predict motif – function associations in any genome, for any given motif.
2. Enabling comparative genomics across large divergences:
  - To apply our pipeline to a relatively less studied species such as *Nasonia vitripennis* (henceforth called the “target” species or genome), we first need to specify the input set of TF motifs. We obtain this from the nearest genome (*D. melanogaster* in this case) where such a collection exists [13], [25]–[26]. (Other taxonomical groups with relatively large collections of experimentally characterized motifs include yeast [14], mouse [13],[27] and human [13],[28].)
  - We consider the possibility that a motif characterized in one species (*D. melanogaster*) may not be usable in a greatly diverged target species (*N. vitripennis*) due to a significant change in the binding specificity of the TF. We address this potential problem by using an automated pipeline to align DNA-binding domains of orthologous TFs in the two species and exploiting structural information to determine if DNA-contacting residues have changed, thereby obtaining information on the evolutionary conservation of the corresponding motifs.
  - The steps outlined thus far (steps 1, 2a, 2b above) are sufficient to discover motif – function associations in the target genome. However, with the goal of boosting the specificity of such predictions, we apply the pipeline separately to the target genome as well as other genomes where the associations are expected to be conserved by and large. We then identify associations that are statistically significant in every species, thus using evolutionary conservation as a “filter”. Unlike previous studies [3],[15],[29] that used the conservation filter to improve binding site prediction (by requiring that sequence alignments reveal the site to be conserved), we use evolutionary conservation at a higher level that does not rely upon non-coding sequence alignment.

Furthermore, we systematically assess the effect of using cross-species comparison on the accuracy of motif function characterization. For this purpose, we design benchmarks comprising highly reliable motif – GO term associations, based on the wealth of chromatin immunoprecipitation (ChIP)-based and genetics-based data on TF – DNA binding in *Drosophila*. We then show that using the new approach (step 2c above) consistently achieves significantly greater precision than the single-species version of the same pipeline.

Applications of pipeline: We first compile a compendium of highly significant motif associations with function categories in GO, through direct application of the above pipeline to *Nasonia*. We then present alternative ways in which comparison of motif – function associations across species can be used to gain biological insights: (a) associations with social behavior-related gene sets in the honeybee are compared with the solitary taxa *Nasonia* and *Drosophila*, in search of a *cis*-regulatory code of sociality, and (b) motifs with known roles in regulation of oxidative phosphorylation in *Drosophila* are tested for associations with this pathway in *Nasonia*.

### A computational pipeline for charting a “motif function map”

**Figure 1:**
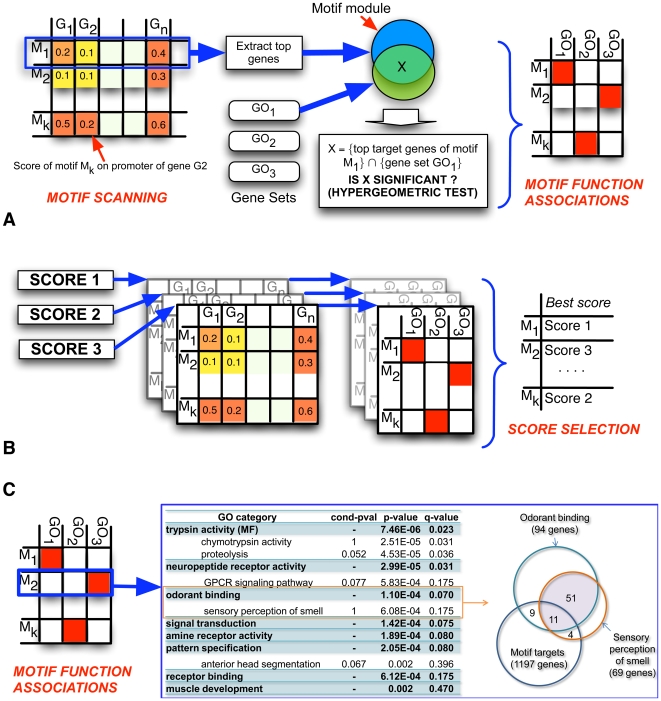
Computational pipeline for charting a motif function map. (A) Each motif is scored against each gene's promoter (“motif scanning”). The top scoring target genes of a motif (“motif module”) are analyzed for enrichment for GO gene sets using the Hypergeometric test, and statistically significant motif – GO associations (red cells) from the test constitute a “motif function map”. (B) Different motif scanning methods produce different motif function maps by the process in (A). For each motif, the best motif scanning method (score) is selected by evaluating each motif function map based on the number of associations and a suitable control (see Methods). (C) For each motif, redundant GO associations are identified by using an extended Hypergeometric test (see Methods) and the motif function map is reorganized. This panel shows GO associations of the Fushi tarazu (FTZ) motif, with redundant associations being indented. The “cond-pval” column is the conditional p-value of an association given the stronger association it is redundant with (see Methods). For example, the association with “sensory perception of smell” is highly significant (p-value∼6E-4), but is “statistically explained” by the association with “odorant binding” (conditional p-value∼1); the Venn diagram on the right illustrates why this is the case.

A “motif module” [5] is the set of genes computationally predicted as being targets of a given motif. A motif module can be tested for statistical enrichment for any given gene set, typically a Gene Ontology (GO) functional category, and the full compendium of statistical associations between motifs and functions is called a “motif function map” [2]. This section describes our new computational pipeline for charting a motif function map. The description follows the outline presented above and is illustrated in Figure 1.

#### Motif scanning methods

The pipeline implements three different motif scanning scores, where a motif is represented as a position weight matrix (PWM) [30].

“site-LLR”: The traditional approach to motif scanning is to find strong matches to the PWM using information theoretic measures of similarity and a high threshold on the similarity measure. The most popularly used binding site prediction programs applicable to single species data belong to this genre (e.g., Patser (http://ural.wustl.edu/software.html) and MATCH [23]). In some cases, a count of such strong matches in a small window (∼500 bp) has been used [22]. We refer to this approach as the “site-LLR” method, and implement our own version as described in Methods.

“Stubb”: In our previous work [2], we argued for the use of a new probabilistic score, obtained from the “Stubb” program [24] based on a hidden Markov model (HMM), that integrates all potential sites, weak and strong, in a small window (∼500 bp), rather than relying only on strong sites defined by *ad hoc* thresholds. The Stubb program computes the likelihood of the sequence under a “two-state HMM” (Figure S1) parameterized by the given motif and then uses its ratio to the likelihood under a null (“background”) model that does not include the motif. This approach is similar in spirit to some other available motif scanning methods, such as “Clover” [7], while substantially different from the site-LLR approach outlined above.

“SWAN”: In order to address certain limitations of Stubb and other existing HMM-based scores (see Discussion), we defined the following new score for motif scanning:

- In the first step, the two-state HMM (Figure S1) is trained on the background sequences, which may be the entire genome, or some selected portion of it. This step learns (via likelihood maximization) a value for the motif transition probability, also called “motif weight”, that captures the frequency of occurrence of the motif in background sequences. Note that “occurrence” here refers implicitly to stochastic transitions to the motif state, rather than to threshold-based matches.
- The second step computes a log likelihood ratio (LLR) score for the target sequence, where (1) the denominator is the likelihood of the target sequence under a new background model – a two-state HMM with motif weight fixed at the value learned in the previous step, and (2) the numerator, as in Stubb, is the likelihood under a two-state HMM with motif weight being a free parameter (constrained to be greater than the motif weight learned above).

We have implemented this new score that we call “SWAN” (**S**tubb **W**ith **A**nother **N**ull) (see Methods for more details, especially with respect to the “background state” in the HMM). Each of the above scores may be used to report the “motif module” for a given motif, as the genes with the highest scoring promoter regions in the genome (Figure 1A and Methods).

#### Motif – function associations

The next step is to search for statistically significant associations between motifs and GO function categories, based on the overlap between a motif module (reported by any of the three scores described above) and the genes in a GO category, using the Hypergeometric test (Figure 1A). This step reports the associations in ascending order of p-values, along with q-values [31] for multiple hypothesis correction. However, it is common for the set of significant associations to include multiple GO categories that are highly overlapping/redundant; e.g., a motif may show strong associations with “pattern formation”, and “anterior posterior pattern formation”, the latter being a strict subset of the former. To identify such redundancies in the list of associations, we also produce a reorganized list where, if an association is “statistically explained” by another association already reported (that has stronger p-value), the former is grouped with the latter to distinguish it from a truly distinct association. Figure 1C shows a snapshot of this reorganized format for reporting associations. We quantify the notion of one association “statistically explaining” another association by extending the Hypergeometric test to consider three subsets instead of the usual two, and imposing the observed overlap structure of these sets as a constraint that the computed p-value is conditional on (see Methods for details, and also see Discussion for related work, e.g., Grossmann et al. [32]). A similar reorganization is also applied to reports of all motif associations for any particular GO category; this is important since our motif compendium includes multiple motifs for the same TF, and also because in some cases different TFs have very similar binding specificities.

#### Selection of motif scanning method for each motif

The computational pipeline includes three motif scanning methods, “site-LLR”, “Stubb”, and “SWAN”. One of these methods is to be used to predict the motif module required for detecting motif – function associations. However, it is not clear *a priori* which method would be most suited to this goal, or whether any one of these methods would be the best choice for all motifs. The next component of the pipeline selects the best motif scanning score for each motif (Figure 1B), by evaluating the motif function map arising out of each score on a genome that is not the target genome and where GO annotations for genes are available (*Drosophila* in our case). The selected score will now be used in applications of the pipeline to the target genome.

**Figure 2:**
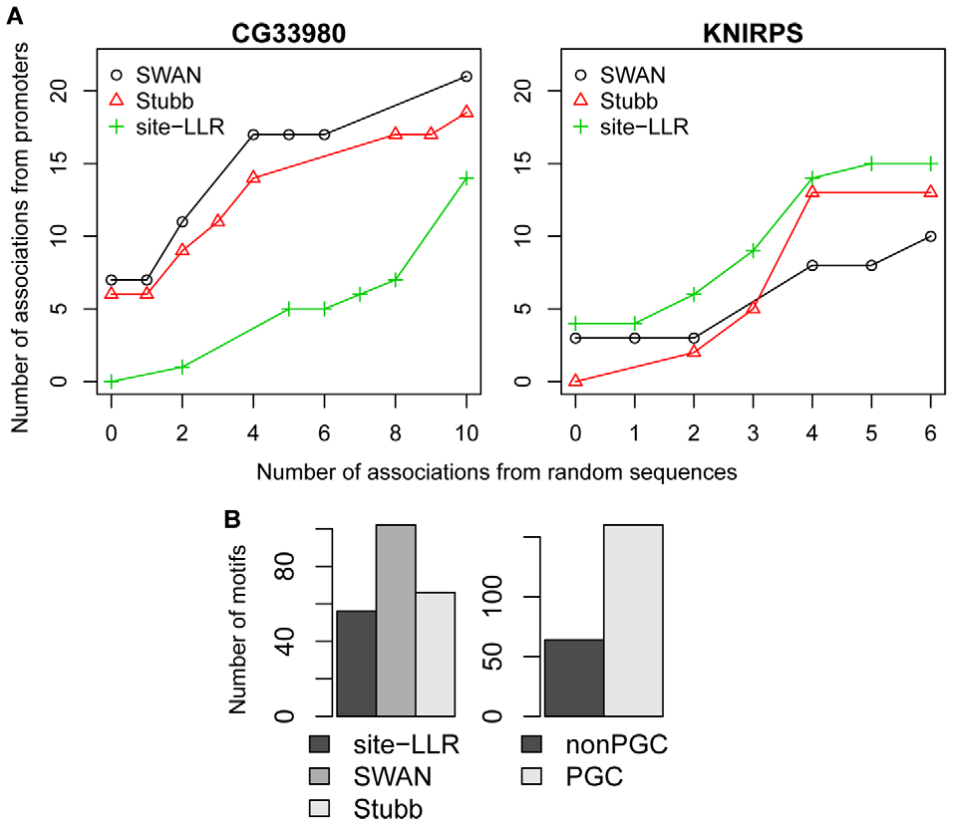
Comparison of motif scanning methods. (A) Two examples of how different motif scanning methods were compared, corresponding to two different motifs (“CG33980” and “KNIRPS”). The y-axis plots the numbers of associations between the motif and the real promoter set, at different levels of significance (always with p-value<0.05), and the x-axis shows the number of associations with the shuffled promoter set at the same level of significance. In both examples, there is a method that is superior by all three measures used for comparison: “strong criterion”, “AUC” and “N0” (see Methods). For KNIRPS, while site-LLR performs best, Stubb dominates SWAN by the “AUC” measure, SWAN dominates Stubb by the “N0” measure (the value of y at x = 0), and there is ambiguity in terms of the “strong criterion”. (B) Comparison of different motif scanning methods, using the number of motifs for which each method performed best as per the AUC criterion. Left panel: comparison of site-LLR, SWAN, and Stubb. Right panel: evaluating the effect of “PGC” parameter (see Methods).

The evaluation is based on the following simple premise: (1) the better motif scanning method should lead to more associations (at the same statistical level of confidence) between motif modules and GO categories, and (2) if we randomize (shuffle) each gene promoter, the recomputed motif modules should not have significant associations with GO categories. While it is clear that the second condition serves as a form of “negative control”, its precise motivation may not be obvious at first. A significant p-value of association between a motif module and a biological gene set is a potentially interesting finding, *provided* that the motif module consists of sequences specifically associated with the motif (TF). This requirement may not always be met, for example if an unusual nucleotide composition (G/C content) of the promoters of a gene set leads to several false binding site predictions and therefore to a false motif association. This phenomenon was widely observed in our previous analysis of the *Apis mellifera* genome [33]. The second condition defined above explicitly tests for such false associations that are artifacts of abnormal G/C content rather than reflecting enrichment for the motif pattern. Details of our evaluation scheme are described in Methods (see Figure 2A for two example evaluations).

Based on evaluations in the *Drosophila* genome, we found that different motif scanning programs perform best for different motifs (Figure 2B). Of the 224 motifs in our compendium, SWAN, Stubb, and site-LLR were the best method (by the “AUC” criterion, see Methods) on 102, 66 and 56 motifs respectively (see Figure S2 for comparisons by other measures). We next asked if certain motif characteristics (e.g., G/C content, length, information content) were correlated with amenability to specific methods (Table S1). The only such correlation observed was that Stubb tended to be especially suited to motifs with low G/C content (p-value <0.01). We also used this evaluation approach to choose important parameters for the methods (see Methods and Figure 2B).

### Extending the pipeline to use information from other species

#### Motifs and GO annotations

**Figure 3:**
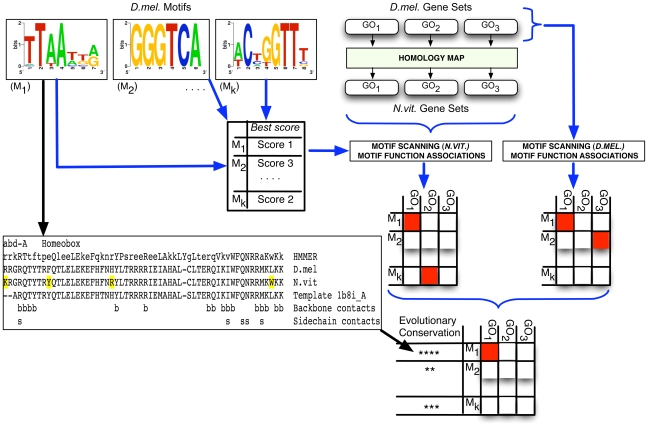
Extended computational pipeline to use information from other species. Motifs and GO annotations were collected from *Drosophila* (“*D.mel.* Motifs” and “*D.mel.* Gene Sets”), and the best motif scanning score for each motif was obtained as described in Figure 1(C). GO annotations in *Nasonia* (“*N.vit.* Gene Sets”) were obtained from the *Drosophila* gene sets using a “homology map” for the two genomes. Motif scanning was performed using the selected scores, followed by motif function map construction in each genome separately. Motif – GO associations that were statistically significant in both species were reported, along with information on evolutionary conservation of the motifs. An example of how motif conservation was investigated is shown in the bottom left panel. The homeobox domain of the transcription factor ABD-A was identified in *Drosophila* and *Nasonia* using HMMER (row 1), the orthologous domains in the two species were aligned (rows 2 and 3), and a similar domain from the PDB database was added to the alignment (row 4). The positions marked in yellow are where amino acid substitutions were seen, but none of these coincides with positions of DNA-contact (rows 5 and 6) as revealed by the structural template, suggesting that the DNA-binding specificity of ABD-A is conserved (“four stars”, for MCS = 4) between the two species.

In order to apply the computational pipeline to a target genome where motifs and GO annotations are not available, we propose obtaining such data from the nearest genome where they are available. The latter is called the reference genome. (Also see Discussion.) GO annotations are deduced based on a homology map between the target and reference genomes. Motifs from the reference genome are used in the target genome “as is” (Figure 3). However, since the two genomes may be greatly diverged, the pipeline attempts to determine whether a motif from the reference genome is likely to represent the binding specificity of the orthologous TF in the target genome. We used the software of Morozov and Siggia [34] to compare the relevant DNA binding domain (amino acid sequence) in the target genome with its ortholog in the reference genome, employing a structural template (of protein bound to DNA) to identify DNA-contacting residues as the key residues for site recognition. (For domains of the zinc finger family ZF-C2H2, we focused instead on four key residues known to be involved in binding specificity [35].) We then assigned a “motif conservation score (MCS)” to the motif based on whether these key residues were conserved (either identical or changed to a chemically similar amino acid) or not (see Figure 3 and Methods). The reported motif function map indicates whether a motif is evolutionarily conserved in this sense, thereby increasing the reliability of that motif's associations. Among 160 *Drosophila* motifs scored by us, 80% scored highly (MCS ≥3 on a scale of 1 to 4) for conservation in *Nasonia*. Sixty four (28% of all) motifs could not be evaluated by our pipeline due to reasons explained in Table S2.

#### Evolutionarily conserved motif – function associations

Comparative genomics has played a key role in curbing false positive errors in *cis*-regulatory analyses [15]–[16],[36]. However, when the target genome's non-coding part is not alignable with any available genome, most of the existing frameworks for sequence-level comparative genomics are rendered useless. Alignment-free approaches have been proposed to address this problem, in the context of *ab initio* motif discovery [37]–[38], as well as motif target prediction [20]. Here, we exploit the power of comparative genomics by looking for conservation at a higher level, i.e., by finding motif – function associations that are statistically significant in multiple species, even though the motif scanning step is performed independently in the different genomes. We propose applying the pipeline not only on the target genome, but also on one or more other genomes (separately), and reporting motif – function associations that are statistically significant across genomes, based on a “combined p-value” (see Methods) computed from the individual p-values in each genome. For instance, we report below the motif associations that are conserved in *Nasonia* and *Drosophila* (also see Figure 3).

**Figure 4:**
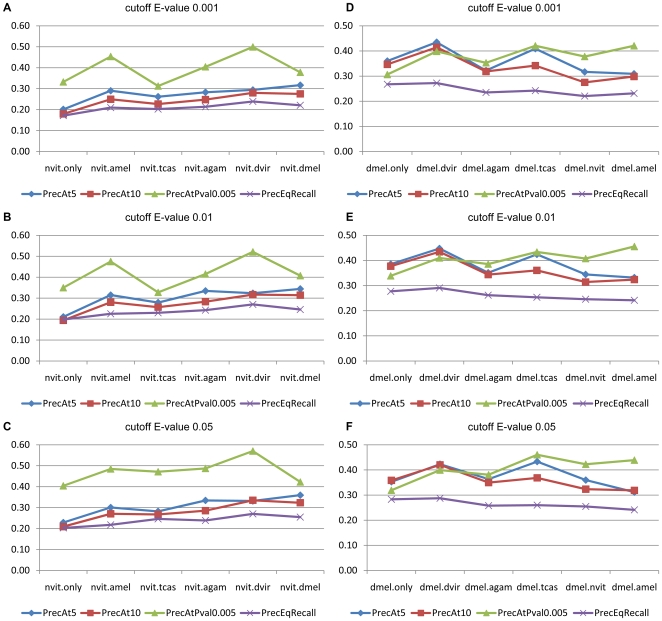
Performance of predicted motif – GO associations using cross-species comparison, evaluated based on ChIP-based binding data. The prediction performance is shown as the precision of the top 5 and top 10 predictions per motif (“PrecAt5” and “PrecAt10”, respectively), the precision at a significance threshold (p-value) of 0.005 (“PrecAtPval0.005”), and as the point where precision equals recall (“PrecEqRecall”). Three different levels of significance (“cutoff E-value” 0.001 (A, D), 0.01 (B, E), and 0.05 (C, F)) were used to define the set of true associations, and the effect of cross-species comparison on the *Nasonia* (A–C) and *Drosophila* (D–F) motif function maps were reported separately.

To assess the advantage of this strategy, we constructed a benchmark of highly reliable motif – function associations that were based on chromatin immunoprecipitation (ChIP)-based TF occupancy data. We roughly followed the methodology of Boden and Bailey [39], where a “gold standard” of TF – GO associations was constructed for yeast and human. We started by compiling 13 data sets of ChIP-based binding data in *Drosophila*, corresponding to 10 distinct TFs. We used the respective author-defined TF target gene sets, and compiled the GO terms enriched in these target sets at three different levels of significance (E-value 0.05, 0.01, 0.001, see Methods). These TF – GO associations were treated as the benchmark of “true” motif – function associations that our pipeline would try to predict, either in its single species version, or by exploiting cross-species comparison. To examine the effect of the species with which comparisons are made, we included the genomes of *Apis mellifera*, *Tribolium castaneum*, *Anopheles gambiae*, and *Drosophila virilis*, in addition to *Drosophila melanogaster*, as separate evolutionary filters for the *Nasonia* motif function map. The performance of these predictions is shown in Figure 4, as the precision of the top 5 and top 10 predictions per motif (“PrecAt5” and “PrecAt10”, respectively), the precision at a fixed significance threshold (p-value of 0.005) (“PrecAtPval0.005”), and as the point where precision equals recall (“PrecEqRecall”). Here, “precision” is the fraction of predicted associations that are “true” and recall is the fraction of “true” associations that were predicted as being significant. We note that the performance (by all measures) improves substantially in going from *Nasonia* (single species) to pairwise comparison-based predictions, the only (minor) exception being the “PrecAtPval0.005” measure for *Nasonia – Tribolium* comparisons (Figure 4A–C). The improvement is most pronounced for *Nasonia* – *Drosophila* comparisons (e.g., “PrecAt5” improves from 0.2 to 0.36), presumably due to the benchmark being from *Drosophila*. We also note that in these large divergence comparisons, the actual evolutionary distance from *Nasonia* (e.g., ∼180 Myrs for *Apis* and ∼300 Myrs for *Anopheles*) does not make a significant difference in performance, except for the “PrecAtPval0.005” measure that is substantially more improved with *Apis* comparisons than with *Tribolium* or *Anopheles* comparisons. The effect of cross-species comparison on the *Drosophila* motif function map (Figure 4D–F) shows a slightly different trend. The precision consistently improves in going from *Drosophila melanogaster* (single species) to *Drosophila melanogaster – Drosophila virilis* comparison-based predictions, although the recall drops (“PrecEqRecall” remains at 0.30 in either case). However, comparison with largely diverged species such as *Anopheles*, *Tribolium*, *Nasonia* and *Apis* suffers both in precision and recall, again with the exception of the “PrecAtPval0.005” measure which conveys a mixed message. Finally, we observe that single species predictions are substantially better in *Drosophila* than in *Nasonia*, which is expected since (a) the benchmark associations are derived from *Drosophila* and may not be biologically “true” in *Nasonia*, and (b) the pipeline's application to *Nasonia* uses motif and GO data from *Drosophila*. The above trends, and particularly the improvement in precision through the use of cross-species comparison, were also confirmed with a second benchmark that we constructed based on *bona fide* TF binding sites from the REDfly database [25]. (See Methods and Figure S3.)

### Applications of computational pipeline

#### Motif function map in *Nasonia*, *Apis*, and *Drosophila*, based on Gene Ontology

The pipeline was run on *Drosophila* with the score selection component (Figure 1B) activated, and then run on *Nasonia* and *Apis* with the scoring scheme selected for each motif. We used a collection of experimentally validated motifs in *Drosophila* obtained from various sources (see Methods). An online interface to the motif function map in each of the three species is available at http://europa.cs.uiuc.edu:8080/nasonia/. For each species, this provides a “motif-centric view”, i.e., all GO associations for each motif, and a “function-centric view”, i.e., all motif associations for each GO category.

#### Motif function associations common to *Nasonia* and *Drosophila*

**Figure 5:**
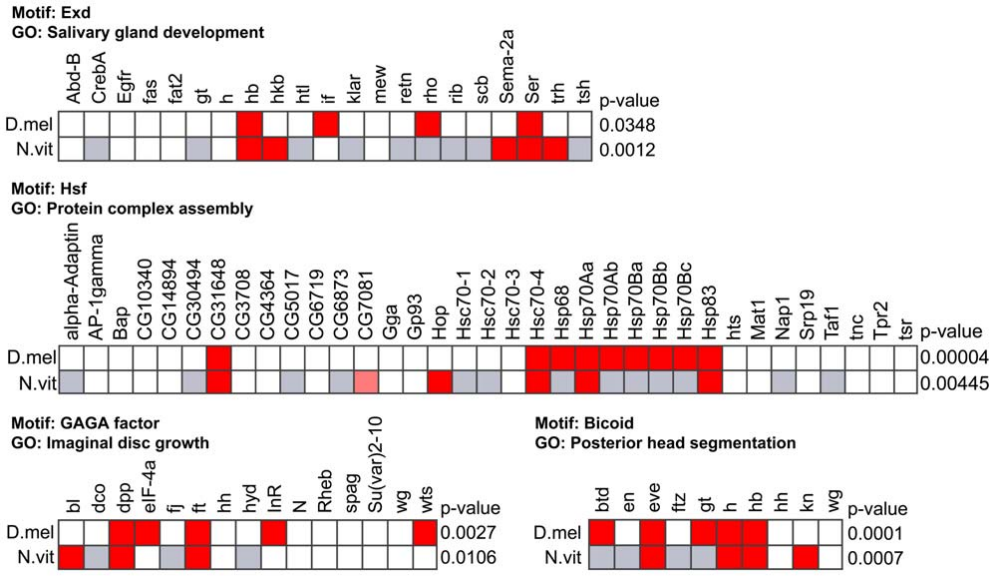
Example of conserved motif – GO associations between *Drosophila* and *Nasonia*. All genes in a GO category in *Drosophila* are shown as columns, with grey indicating that the ortholog was not found in *Nasonia*. Dark red indicates motif presence at the threshold used, light red indicates medium strength motif presence, white indicates motif absent in the gene's promoter. The p-value of association between motif and GO category is shown for each species, at the right end of its row.

We looked for motif – function associations that were statistically significant in both *Nasonia* and *Drosophila* based on combined p-values. Overall, 177 such associations were discovered at a q-value of less than 0.05, representing evolutionarily conserved and presumably more reliable associations (Table S3). (All such associations had uncorrected p-value <0.004.) 91 of these 177 associations were non-redundant, 119 (67%) were for motifs that were scored for evolutionary conservation and 99 (83%) of these were highly conserved (motif conservation score MCS ≥3 on a scale of 1 to 4), as reported in Table S3. (MCS of 3 or more implies that every critical residue in the DNA-binding domain is either exactly conserved or substituted by an amino acid with a similar biochemical characterization (see Methods).) The discovered associations included several regulatory interactions that have already been experimentally characterized, chiefly in *Drosophila*. For instance, the motif for Suppressor of Hairless (SU(H)) is associated with the GO category “Notch signaling pathway” in both species (combined p-value 5E-9, *Drosophila* p-value 4E-6, *Nasonia* p-value 9E-5); the role of SU(H) in regulation of this pathway is well known [40] and conserved even in vertebrates [41]. The motif for Abdominal B (ABD-B) (MCS = 4) is associated with “ectoderm development” (p-value 1E-5, supported by [42]) and “salivary gland development” (p-value 7E-5, supported by [43]). The GAGA motif was assigned to several different biological processes, e.g., “tracheal system development” (p-value 4E-6) and “mesoderm development” (p-value 9E-6), consistent with its previous characterization as potentially regulating a broad range of cellular processes [44]. Some of the other most significant motif – function associations that are supported by the literature include Hunchback (HB) with “nervous system development” (p-value 5E-7, supported by [45]), Zerknullt (ZEN) (MCS = 4) with “ectoderm development” (p-value 3E-5, [46]–[47]), Mitochondrial transcription factor A (MTTFA) with “apoptosis” (p-value 3E-5, [48]), Antennapedia (ANTP) (MCS = 4) with “antennal morphogenesis” (p-value 0.001, [49]) and with “central nervous system development” (p-value 6E-5, [50]), and Heat shock factor (HSF) (MCS = 2) with “response to heat” (p-value 4E-5, [51]), among others. (The low MCS of HSF is due to a single substitution (I→M) at a predicted backbone contact residue, see Table S4.) Figure 5 shows, for four examples of conserved motif association, the motif targets and non-targets in both species.

We also found conserved motif – function associations that have not been previously identified in any species. For example, Reversed polarity (REPO) (MCS = 4) is associated with “transmission of nerve impulse” (p-value 4E-5). REPO is a major player in glial differentiation [52] and may be involved in transmission of signals [53].

In some cases, conserved motif associations could not be unambiguously assigned to a TF, due to similar binding specificities of different TFs. The Sex combs reduced (SCR) motif (MCS = 4) was assigned to the functional category of “proteolysis” in both species; however this association was statistically explained by associations for similar motifs Empty spiracles (EMS) (in *Drosophila*) and Buttonless (BTN) (in *Nasonia*, MCS = 3), and our discovery may be pointing to an enrichment for a homeobox motif [T/C/A]TAAT[G/T][A/G] in the promoters of proteolysis-related genes, rather than for SCR binding sites in particular.

The CG12361 motif (MCS = 4) was associated with “cyclic nucleotide metabolism” (p-value 4E-5). This motif targets 7 of the 26 genes in this GO category in *Drosophila* and 6 of the 19 genes in the orthologous set in *Nasonia*, but the target sets (of size 7 and 6 respectively) are mutually exclusive. This presents an interesting situation where motif – function associations are conserved but our procedure does not find the corresponding motif – gene associations to be evolutionarily conserved. In other words, the association is discovered only at a higher level of comparative genomics.

The biological process “ectoderm development” was found to be associated with 15 non-redundant motifs (combined p-value<0.004, q-value<0.02), indicating that this is in part a highly conserved transcriptional network. Many of these associations are for motifs of factors with known roles in this process (e.g., ABD-B (MCS = 4) [42], ZEN (MCS = 4) [46]–[47], Abdominal A (ABD-A) (MCS = 4) [54], GAGA factor [55], SCR (MCS = 4) [56], Odd skipped (ODD) [57], ANTP (MCS = 4) [58]), while others are (to our knowledge) novel associations not reported in the literature (Extradenticle (EXD) (MCS = 4), HB, CG7056 (MCS = 2), PDHP (MCS = 4), Bric a brac 1 (BAB1), Hairy (H) (MCS = 4), SU(H)). The predicted motif change for CG7056 was due to two substitutions at key residues, and was corroborated by a specialized tool (http://ural.wustl.edu/flyhd/) that predicts the specificity of homeodomains (Figure S4).

The motif for the TF Bicoid (BCD) was found to be associated with “posterior head segmentation” and “trunk segmentation”. This is interesting because BCD is associated with segmentation in *Drosophila* [59], but there is no known ortholog of this factor's gene in *Nasonia*. The apparent conservation of the BCD – segmentation association may be due to another motif (Orthodenticle (OTD)) that is very similar to the BCD motif and is believed to play an important role in the above biological functions [60]. We discuss later the confounding effect of multiple TFs with similar binding specificity, as in this example, and its implications for our analysis.

Searching for motif associations that are statistically significant in the target genome (*Nasonia*) and another genome (*Drosophila*) is not the only manner in which evolutionary comparisons can inform a motif function map. In the following two subsections, we illustrate alternative ways in which cross-species comparisons may lead to new biological insights. A third example analysis is presented in Supplementary Text S1.

#### Motifs associated with social behavior in honeybee: *Nasonia* as an evolutionary filter

First, we present an example analysis where evolutionarily conserved associations may be of lesser interest biologically than lineage-specific ones. The honeybee, *Apis mellifera*, is a model organism for studying social behavior, and prior work has identified gene sets whose expression in the brain responds to social cues during behavioral maturation (e.g., from nurse to forager bees [61]). *Nasonia* is a member of the Hymenoptera order, to which the honeybee also belongs, but is not a social animal. Therefore, a motif association that is specific to behavioral gene regulation in *Apis* should be absent in *Nasonia*, when considering *Nasonia* orthologs of the same gene sets. Likewise, a conserved motif association undermines the hypothesis of a social behavior-specific role, and is likely an artifact of a more basal (not sociality-specific) biological process that these genes are part of in both species. Working with gene sets analyzed in [33], we identified significant motif associations in *Apis*, and noted also the p-values of association (of the same motifs) from orthologous sets in *Nasonia* and *Drosophila*. Genes up-regulated in the *Apis* brain in response to Manganese treatment showed 67 significant motif associations in *Apis* (Table S5). However, upon invoking an “evolutionary filter” that requires the p-values in the other two (asocial) species to be *above* a threshold, only 27 associations remained. Thus, for this gene set, cross-species comparison was able to filter out 40 (59%) of the *Apis* associations (31 due to the *Nasonia* filter).

We also found 14 motif associations for other social behavior-related gene sets from *Apis*, four of which (including the previously predicted role of the GAGA factor [33]) do not pass the evolutionary filter (Table 1). The remaining ten (9 distinct) associations are potentially involved in the social regulation of gene expression in honeybee brains. Particularly interesting are the bee – specific enrichment for Broad (BR), Adh transcription factor 1 (ADF1) and Tramtrack (TTK) motifs in gene sets responding to Methoprene treatment – Methoprene is a Juvenile Hormone analog that causes precocious foraging behavior, the TF BR is known to respond to hormone stimulus [62], ADF1 is known to be involved in memory, learning and certain behaviors in *Drosophila* [63], and TTK is known to have mutant phenotypes affecting aggressive behavior [64]. (None of these motifs could be scored for evolutionary conservation.) The association between H (MCS = 4), a factor involved in sensory organ development (a Juvenile Hormone dependent process [65]), and a set of genes over-expressed in foraging bees (also a Juvenile Hormone dependent condition) [61],[66] is also notable (Table 1). The motifs involved in some of the other bee – specific associations are known to play important roles in nervous system function and development in *Drosophila*, e.g., Mothers against dpp (MAD) (MCS = 4) is known to regulate synaptic growth [67], and Knirps (KNI) is known to be involved in dendrite morphogenesis [68].

**Table 1:** Motif associations with gene sets implicated in social behavior in honeybees.

Gene set	Motif	MCS^a^	Motifsource	A.mel	N.vit	D.mel
Pre-foraging maturation↑ (top 100)	Trl	?	F	**0.001** *	**0.066** ***	0.254
Methoprene↓	I_ADF1_Q6	?	T	**0.001** *	0.155	0.569
Forager↑	hairy.new.6	4	B	**0.003** *	0.650	0.870
Pre-foraging maturation↑ (top 100)	I_GAGAFACTOR_Q6	?	T	**0.004** *	**0.039** **	0.654
cGMP↑	Kruppel	2	T	**0.005** *	0.217	**0.046** **
Methoprene↑	Ubx.txt	4	F	**0.008** *	0.767	0.855
Methoprene↓	Adf1	?	F	**0.008** *	0.604	0.951
cGMP↑	Kr	2	F	**0.008** *	0.304	**0.016** **
cGMP↑	kni	4	F	**0.008** *	0.922	0.107
Methoprene↓	br-Z3	?	F	**0.008** *	0.286	0.895
Hive bee to forager transition↓ (top 100)	CG11085.new.7	?	B	**0.009** *	0.397	0.577
Pre-foraging maturation↑ (top 100)	CG7056.new.7	2	B	**0.009** *	0.905	0.643
Hive bee to forager transition↓ (top 100)	Mad	4	F	**0.010** *	0.701	0.561
Methoprene↑	ttk.new.6	?	B	**0.010** *	0.733	0.742

Gene sets (column 1) are from [33], associated motifs are listed in column 2, and the p-value of association in *Apis*, *Nasonia* and *Drosophila* are listed in columns labeled “A.mel”, “N.vit” and “D.mel” respectively.

a Motif conservation score.

*: p-value<0.01.

**: 0.01≤ p-value <0.05.

***: 0.05≤ p-value <0.1.

Motif source: B, B1H; F, flyreg.org data; T, Transfac.

#### Oxidative phosphorylation genes in *Nasonia*

In this analysis, we show how predicted motif associations in the target genome may be substantiated by literature-based evidence from another species, rather than by statistical significance of the same association in the other species. The oxidative phosphorylation (OXPHOS) pathway plays a very important role in the production of ATP, the principal source of cellular energy. Evolution of OXPHOS genes, their structure, and regulation, has been studied previously [69], and annotation of genes from this pathway in the *Nasonia* genome offers the opportunity to extend our understanding of this key pathway. We analyzed the promoters of 58 annotated *Nasonia* OXPHOS genes [70]. We first compiled a list of ten known motifs from studies of OXPHOS regulation in fruitfly and mammals (in some cases, the fruitfly homolog of the mammalian element was used). These are: DNA replication-related element factor (DREF), Erect wing (EWG) (*Drosophila* homolog of NRF-1), Buttonhead (BTD) (*Drosophila* homolog of SP1), DATF-2 (*Drosophila* homolog of CREB/ATF2), Pleiohomeotic (PHO) (*Drosophila* homolog of YY1), AP1 (related to NRF-2), E-BOX, OXBOX, PR1, and the Nuclear Respiratory Gene element (NRG) [69], [71]–[74]. Significant associations (p-value<0.05, q-value<0.08) were found for four of these ten motifs: BTD (10/58 genes targeted), AP1 (9/58 genes targeted), PHO (9/58 genes targeted), and PR1 element (9/58 genes targeted) (Table 2). When we repeated the above analysis with all motifs in our collection (Table S6), these four motifs came out as the four strongest associations overall. We note that none of the four above-mentioned motif associations achieved statistical significance with combined p-values from *Nasonia – Drosophila* comparisons. Thus, while this analysis predicts a role for the motifs BTD, AP1, PHO and PR1 in OXPHOS regulation in *Nasonia*, it is important here to evaluate the evidence in light of the literature-based support from *Drosophila*.

**Table 2:** Enrichment p-values for the oxidative phosphorylation gene set in *Nasonia*, shown here for ten motifs implicated in the literature as having a regulatory role in this pathway.

Motif	MCS^a^	Motif source	p-value	q-value	#common^b^	#motif targets^c^	#genes in gene set^d^	#total^e^
btd.new.6	?	B	0.0117	0.0776	10	696	58	9097
V_AP1_C	4	T	0.0322	0.0776	9	701	58	9097
pho	4	F	0.0362	0.0776	9	716	58	9097
PR1	?	L	0.0461	0.0776	9	749	58	9097
nrg	?	L	0.2491	0.3355	6	666	58	9097
dATF2	?	L	0.3744	0.4202	5	630	58	9097
Ewg	?	L	0.4557	0.4323	5	693	58	9097
I_DREF_Q3	?	T	0.5135	0.4323	5	739	58	9097
OXBOX	?	L	0.6496	0.4861	3	518	58	9097
Dref	?	F	0.8672	0.5316	3	749	58	9097
E-box	?	L	0.8684	0.5316	3	751	58	9097

Motif source: B, B1H; F, flyreg.org data; T, Transfac; L, literature.

a Motif conservation score.

b Number of genes common in motif target and oxidative phosphorylation genes.

c Number of motif target genes.

d Number of genes in oxidative phosphorylation gene set.

e Total number of genes in the analysis.

## Discussion

Our work explores the following challenging question related to comparative regulatory genomics: *how can we “import” the wealth of molecular information in well-studied genomes such as Drosophila to the regime of a less studied genome such as Nasonia, given that the non-coding regions of the genomes do not align?* We address this question in the context of characterizing motif functions. We identify several methodological issues involved here, and present a computational pipeline that incorporates novel solutions to the issues. Our approach is expected to become increasingly relevant as hundreds or even thousands of other metazoan genomes get sequenced in the future.

### Pipeline for motif function map in single genome

We recognize that there are several alternatives to motif scanning that have not been explored here. Boden and Bailey [39] point out that most motif-scanning software can be characterized by two basic ideas: “maximum-odds and hit count” (finding strong sites and counting them) and “average odds” (summing over all possible sites, weak or strong). We believe that our choice of the “site-LLR” and the Stubb/SWAN methods capture the essence of these two popular ideas, and are therefore representative of existing knowledge on motif scanning. Approaches that use additional information such as phylogenetic profiles [75] are not evaluated here, as it is not clear how evolutionary information may be extracted from a genome whose non-coding part is not alignable with other species. It will be also be interesting to examine if more biophysically inspired methods, like TRAP [76], provide complementary strengths in motif scanning, as suggested in recent work by Roider et al. [1]; however, here we chose to operate within the statistical regime of the HMM that has been studied more extensively in the literature.

The log likelihood ratio (LLR) score computed by Stubb and other HMM-based methods [24], [77]–[78] asks the statistical question: *does the motif help “explain” the data (sequence) significantly better than the background model can*? In some cases, this may not be the right statistical question to ask. We illustrate this issue with the example of the “HB” motif (consensus: TTTTTTGTT). This motif has a high match score to the poly-T string (TTTTTTTTT), but this is not only because the motif roughly matches the string, it is also in part because poly-T substrings happen to be more common in the genome than the simple background model can capture. A low order Markov chain that is typically used as background model may be inadequate to capture frequencies of certain patterns (such as the poly-T substring) in the background sequences; if such patterns happen to be similar to a motif, the inadequacy of the background model will adversely affect the LLR statistic used for motif scanning. To address this, the newly defined SWAN method asks the following, different question: “given that we must use a two-state HMM to explain/parse a sequence, *are we significantly better off using a higher motif weight than the value learned from background sequences*?” (Also see [79]–[80] for similar ideas.)

To our knowledge it has never been tested systematically whether some TFs tend to operate mostly through strong binding sites while others frequently make use of strong as well as weak sites and their clustering. Existing work on motif function maps (e.g., [1]–[2],[39]) have each shown that clustering of strong and weak sites is more efficacious than using strong sites alone, when testing with entire compendia of motifs. Surprisingly, past work has not considered the possibility that the choice of motif-scanning method may need to be motif dependent. We examined this issue, and found that each of the three statistical approaches evaluated is clearly the better choice for a sizeable set of motifs.

In the type of analysis presented here, one must keep in mind that the statistical associations are for motifs, and not for TFs *per se*. Different TFs may have very similar binding specificities (motifs) and an association with the motif for TF *A* may in fact be due to TF *B* with similar binding affinity. As such, claims about motif associations may or may not be valid when extrapolated to corresponding TFs. Another limitation of our analysis (and one that is unfortunately common to most genomic studies today) is that multiple hypothesis correction, performed here through the use of q-values, does not account for the fact that the underlying association tests are statistically dependent, often being for highly similar motifs or GO sets. Also, our choice of searching only the 5 Kbp upstream regions of genes may lead to missed sites, but we believe that it results in an overall increase in the signal to noise ratio. Other possibilities, such as including all of a gene's surrounding region up to the neighboring genes on either side, or including intronic regions, provide avenues for future research.

The task of finding statistical associations between gene sets and annotations has itself been a topic of much research, as reviewed in [81]. In particular, several ideas have been presented to deal with redundancies between GO categories. Grossmann et al. [32] deal with “parent-child” relationships present in the GO hierarchy, by modifying the Hypergeometric test. This is closest in spirit to how we handle redundancies among gene sets, except that our approach is designed to work for any pair of gene sets (**E** and **O**, following terminology introduced in Methods), and not only for “parent-child” pairs. This is useful not only to deal with redundant pairs of GO categories (such as “odorant binding” and “sensory perception of smell”, with an overlap of 62/69), but also to deal with pairs of motif modules that are largely overlapping.

### Extending the pipeline to use information from other species

We have demonstrated, on benchmarks constructed from ChIP-based and genetics-based data, that requiring cross-species conservation of motif – function associations leads to significantly higher specificity. While *Nasonia – Drosophila* comparison clearly improved specificity compared to single species analysis on *Nasonia*, we did not see a clear effect of varying evolutionary divergence from the compared species. However, we believe that the reference genome, i.e., where the motif and GO data are “imported” from, is the most judicious choice for cross-species comparison. We also note that our approach is distinct from imposing the conservation requirement at the motif scanning stage, as was done in the alignment-free method of [20]. Our reasoning was that motif – function associations may be “evolutionary robust”, i.e., detectable even though the motif – gene relationships are not detected as being conserved, as illustrated by the discovered association of CG12361 with cyclic nucleotide metabolism.

The choice of the reference genome, for a particular target genome under study, will generally be clear, since the kind of comprehensive molecular data that is required of the reference species is available for only a handful of species. However, we note that the motifs and GO annotations that are “imported” from the reference to the target genome need to be by and large conserved: if the divergence is too great, (a) most motifs will receive low conservation scores and hence be unreliable, and (b) GO gene sets inferred in the target genome will be highly erroneous, leading to very few significant motif associations.

Our approach to characterizing motif conservation levels is only a first step to solve an important problem in comparative regulatory genomics – to use motifs characterized in one species for analyzing the genome of a highly diverged species. Morozov and Siggia [34] have considered this problem for yeast TFs, and have attempted to model the impact of key residue changes on binding specificity. Similar goals have been pursued by Noyes et al. [82] for homeodomain factors in *Drosophila*. For now, our pipeline only uses information on conservation (or substitution to a similar amino acid) to roughly estimate the impact on binding specificity, but future versions will attempt to do this in a more quantitative and sensitive manner.

We also note that functional characterization of a transcription factor may be undertaken in a more direct manner through ChIP-chip or ChIP-seq assays for the factor's binding locations, and may even be coupled with cross species comparison to achieve high specificity. Given the current technology, this approach is clearly more expensive than computational frameworks such as ours, although it can serve as a follow-up to specific motif associations identified computationally.

## Methods

### Sequence data

5 Kbp promoters of *D. melanogaster* (Release 5) and *D. virilis* (Release 1.2) were obtained from FlyBase [83]. *A. gambiae* (Feb. 2003) promoters were downloaded from UCSC Genome Browser Database [84]. Promoters of *A. mellifera* (Amel_2.0), *N. vitripennis* (Nvit_1.0, RefSeq set only – 9163 genes), and *T. castaneum* (Tcas_1.0) were taken from HGSC (http://www.hgsc.bcm.tmc.edu/).

### Motif compendium

A total of 224 motifs were obtained from Transfac [13] (40 motifs), FlyReg [85] (52 motifs), the literature [69],[72],[74] (7 motifs), and from [26] (125 motifs) (Supplementary Text S2).

### Details of SWAN and Stubb motif scanning methods

(a) Raw score of each window was computed as the LLR described in Results. (b) P-value of the window's raw score was computed empirically based on 1000 genomic windows with the same G/C content as the original window. This is referred to as the “PGC” technique below. (c) A gene was declared as a motif target if any window in its 5 Kbp promoter had a p-value below 0.005. “Stubb” scores were computed using the SWAN program and a motif weight of 0. Other details are identical to SWAN. The “background state” in the two-state HMM used by Stubb and SWAN (to score a sequence as well as in learning the motif weight) was set to emit according to single nucleotide frequencies in the sequence under consideration (i.e., a “local background”).

### Details of “site-LLR” motif scanning method

The log-likelihood ratio (LLR) score of a string *s*, given a motif W, is defined as log [Pr(*s* | W)/Pr(*s* | Bkg)], where “Bkg” refers to the background model (same as for SWAN above). Given a motif, we computed the maximum possible LLR score of a site (over all possible sites), and using a threshold equal to 0.9 times this maximum LLR, we marked all sites that were above the threshold. Empirical p-value of a sequence window was computed as per the following ordering: (i) a window with more marked sites scores higher; (ii) if two windows have the same number of sites, the window with the stronger individual site scores higher.

### Defining GO gene sets

The homology map among *Anopheles*, *Apis*, *Drosophila*, *Nasonia*, and *Tribolium* was obtained from http://cegg.unige.ch/. For each *Drosophila* gene in a GO category (from association files published at http://genemerge.cbcb.umd.edu/associationfiles/ in November 2005), all its orthologs in the second species were included in the GO category definition for that species. Thus, a GO category's cardinality may be different in different species.

### Evaluation of methods

For a given motif, its target sequences were determined in the set of real *Drosophila* promoters and in the set of shuffled promoters. Motif modules thus determined were tested for association with GO categories, and a “true positive versus false positive curve” was drawn to plot the number of associations in the real set and in the shuffled set, at different thresholds of significance (see Figure 2A for two example plots). Any two motif scanning methods were compared in the following three ways: (a) “*Strong criterion*”: the curve of one method completely dominates the plot for the other method. (b) “*AUC*”: the area under one curve is greater than that under the other curve. (c) “*N0*”: the number of associations in the real set at a significance threshold where the number of associations in the shuffled set is 0. The following methods and techniques were evaluated:

- Scanning method (site-LLR, Stubb, SWAN).
- “PGC” technique: as mentioned above, the raw score of a particular method on a given sequence window was converted into an empirical p-value of motif occurrence by comparing with scores of 1000 randomly selected windows. We evaluated two different ways to choose these windows: (i) from all non-coding sequence windows with the same G/C-content (“PGC” technique), and (ii) from all non-coding sequence (non-PGC technique). Figure 2B shows that the former was superior on the majority of motifs.

### Multiple hypothesis corrections

Q-values [31] are calculated for each motif, correcting for all GO association tests, as well as for each GO category, correcting for all motif association tests.

### Dealing with redundant associations

Let **M** be a set of interest in the universe **U**, and let **E** and **O** be two other subsets of **U**, with the cardinality of **U**, **M**, **E** and **O** being *N, m, n_1_* and *n_2_* respectively. Let **| M ∩ E | = γ, | M ∩ O | = λ** and **| E ∩ O | = α**. Typically, **E** and **O** will be two sets whose associations with **M** are both statistically significant, and we are interested in asking: *Does the association between* **M** *and* **E** *statistically explain the association between* **M** *and* **O** *in some sense?* The unconditional (traditional) p-value of association between **M** and **O** is given by the probability that a random set of size **| O | =** ***n_2_*** has an overlap of size greater than or equal to **| M ∩ O | = λ** with **M**. We answer the above question by calculating the probability of this event *conditional on the observed overlap cardinality between* **M** *and* **E** *and that between* **E** *and* **O**. In other words, if **R** is a random subset of **U**, with cardinality *n_2_*, we calculate the probability 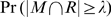 conditional on |R| = *n_2_* and |R ∩ E| = α, where E is a fixed subset of cardinality *n_1_* and |M ∩ E| = γ. This is computed as:
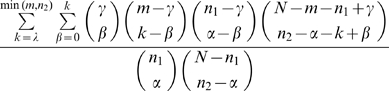
We note that this is an example of the multivariate hypergeometric distribution.

### Combined p-values

A motif was tested for consistent association with a gene set in multiple species as follows. Let *p_1_*,*p_2_*,…,*p_k_* be the p-values of a motif – function association in *k* different species. We first compute the combined statistic:
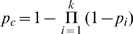
We denote this random variable as 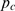 and its observed value as 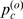. Under the null hypothesis that each *p_i_* is uniformly distributed, we computed the probability π_c_ that the combined statistic 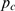 has a value less than or equal to the observed value 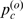, i.e., p-value for the combined statistic *p_c_*, as follows:
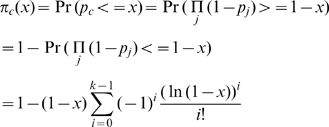
where the last step is due to the fact that 1−*p_j_* is uniformly distributed in [0,1] under the null hypothesis. The random variable 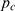 has the desirable property that it is low only if each p_i_ is low, and thus captures consistent motif association (low p-value) in all species. (Contrast this with the product of the *p_j_*'s, which may be low even if one or more of the *p_j_*'s is close to 1.) Note however, the p-value π_c_ computed above corresponds to the strong null hypothesis that every individual *p_j_* is uniformly distributed.

### Assessing motif conservation between *Drosophila* and *Nasonia*

We used an offline version of the tool described in [34]. Starting with the full complement of protein sequences in either genome, this tool first uses the HMMER software version 2.3.2 (http://hmmer.janelia.org/) to scan for matches to DNA-binding domains catalogued in the PFam database [86]. For each TF in *Drosophila*, it then aligns each of its DNA-binding domains to the most similar domain match in *Nasonia*. It then adds to this pairwise alignment a third domain that belongs to the same family and corresponds to a protein whose structure (in DNA-bound state) is available from PDB [87]. By using this (aligned) domain with structural information, the tool identifies DNA-contacting residues (that make either backbone or side-chain contacts) based on a distance threshold. We consider these DNA-contacting residues as the “key” residues. For matches to the zinc finger family (ZF-C2H2), we define key residues to be the four residues identified previously as imparting DNA-recognition capability to this family [35]. Limiting our attention to the key residues only, we then determine if the orthologs from the two species have undergone an amino-acid substitution, and if so, whether the substitution has been to a similar amino acid, as defined by grouping amino acids into one of the following seven classes [88]: (i) amino acids (aa's) with aliphatic R-groups (G,A,V,L,I), (ii) non-aromatic aa's with hydroxyl R-groups (S,T), (iii) aa's with sulfur-containing R-groups (C,M), (iv) acidic aa's and their amides (D,N,E,Q), (v) basic aa's (R,K,H), (vi) aa's with aromatic rings (F,Y,W), and (vii) amino acids (P). Finally, each motif was assigned a conservation score that could take the value “?” or an integer between 1 and 4 (4 for strongest conservation), as per criteria defined in Table S2. The alignments of DNA-binding domains are available in Table S4. Of the 160 motifs that received a motif conservation score (not “?”), 114 (71%) had the highest score of 4, 8% were scored at 3, and 20% received the low score of 2, indicating greater potential for evolutionary change.

### Assessing cross-species comparison

To construct benchmarks based on ChIP data, we collected published target genes for 10 TFs: BCD, Caudal (CAD), Giant (GT), HB, and Kruppel (KR) from [89], Dorsal (DL), Snail (SNA), and Twist (TWI) from [90], gaga factor (GAF) from http://intermine.modencode.org/, and PHO from [91]. Statistically significant motif – GO function associations were identified using the Hypergeometric test and E-value cutoffs (0.001, 0.01, and 0.05). (E-value here is the product of the p-value from the Hypergeometric test and the number of GO terms tested for.) These were treated as the “true” associations, and the associations predicted by the motif function map were evaluated against this benchmark. The following four measures were calculated for each TF and an average over all TFs was computed: (a) “PrecAt5”: precision (number of correct predictions, divided by total number of predictions) when considering the top 5 predicted associations, (b) “PrecAt10”: precision in the top 10 predictions, (c) “PrecAtPval0.005”: precision in the associations with p-value less than 0.005, and (d) “PrecEqRecall”: precision when the number of predicted associations is equal to the number of true associations. The benchmark based on genetics data was constructed similarly with published target genes from the REDfly database [25] except that we used less stringent E-value cutoffs (1, 10, and 50) since the target gene sets here are smaller but more reliable than in the ChIP-based benchmark.

### Supplementary website

5 Kbp promoter sequences, promoter and gene mapping information, motifs, GO gene sets, source code for SWAN, and a link to web interface for a motif function map are available at our site http://europa.cs.uiuc.edu/CompGenomics09/.

## Supporting Information

Figure S1 Two-state HMM of the Stubb program.
(0.20 MB DOC)

Figure S2 Comparison of different motif scanning methods.
(0.09 MB DOC)

Figure S3 Performance of predicted motif - GO associations using cross-species comparison, evaluated based on genetics-based binding data.
(0.27 MB DOC)

Figure S4 Predicted motifs for *Drosophila* protein CG7056-PA and its ortholog in *Nasonia* using the online tool at http://ural.wustl.edu/flyhd.
(0.17 MB DOC)

Table S1 Correlation between motif characteristics and amenability to specific methods.
(0.05 MB DOC)

Table S2 Possible motif conservation scores and their semantics. See text for explanation of how.
(0.03 MB DOC)

Table S3 Motif functional associations common to *Nasonia* and *Drosophila*.
(0.31 MB DOC)

Table S4 Alignments of DNA-binding domains in orthologous protein sequences from *Drosophila* and *Nasonia*.
(1.66 MB TXT)

Table S5 Motif associations with genes up-regulated in response to Manganese treatment in honeybees.
(0.11 MB DOC)

Table S6 Enrichment p-values for the oxidative phosphorylation gene set in *Nasonia*.
(0.05 MB DOC)

Text S1 Chemoreceptor genes in *Nasonia*.
(0.20 MB DOC)

Text S2 224 motifs used in this study.
(0.02 MB TXT)
