# Supplementary figures and images for: Functional Characterization of Transcription Factor Motifs Using Cross-species Comparison across Large Evolutionary Distances
Source: PLoS Comput Biol. 2010 Jan 29;6(1):e1000652. doi: 10.1371/journal.pcbi.1000652 (PMC2813253; doi:10.1371/journal.pcbi.1000652)

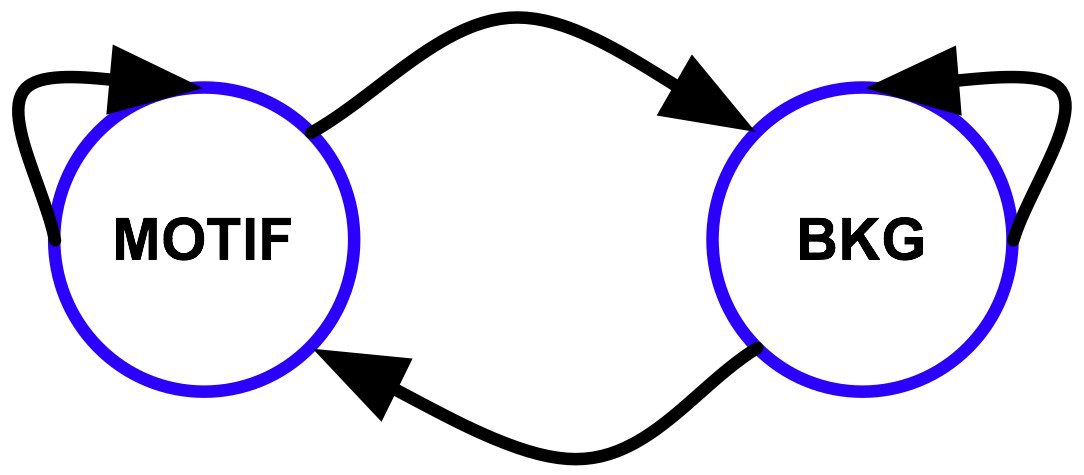


Figure S1. Two-state HMM of the Stubb program.

Supplement: Figure S1 — Two-state HMM of the Stubb program. (0.20 MB DOC) [file pcbi.1000652.s001.doc]
