# Supplementary material for: Functional Characterization of Transcription Factor Motifs Using Cross-species Comparison across Large Evolutionary Distances
Source: PLoS Comput Biol. 2010 Jan 29;6(1):e1000652. doi: 10.1371/journal.pcbi.1000652 (PMC2813253; doi:10.1371/journal.pcbi.1000652)
Supplement: Figure S2 — Comparison of different motif scanning methods. (0.09 MB DOC) [file pcbi.1000652.s002.doc]

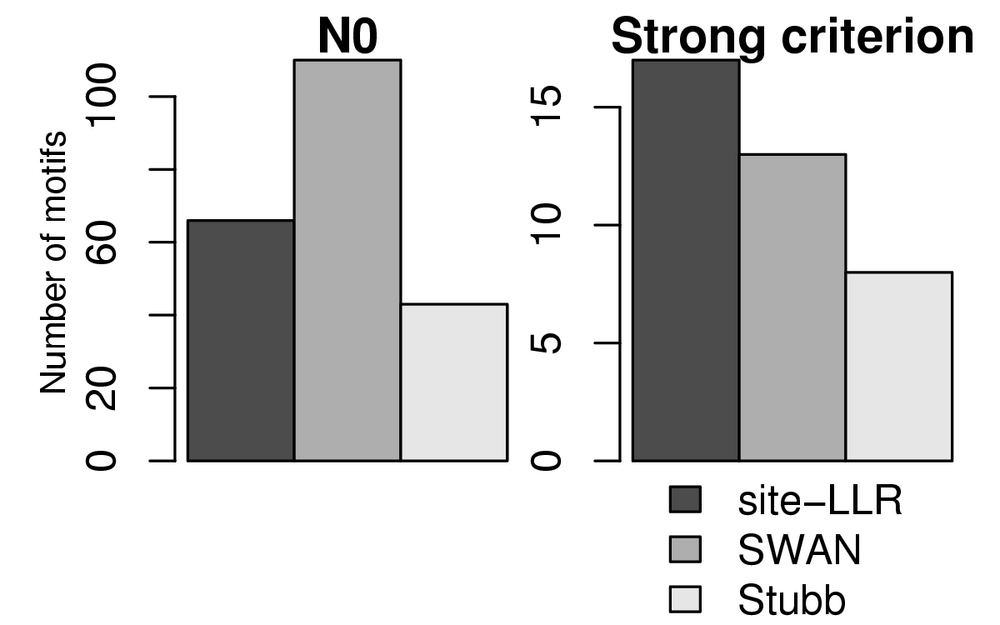


Figure S2. Comparison of different motif scanning methods. The y-axis is the number of motifs (out of 224) on which a method was superior to other methods. Left panel: comparison of methods by “N0” (see Methods section). Right panel: comparison of methods by “Strong criterion” (see Methods section). Tests were done using the “PGC” technique with each motif scanning method, see Methods section). Note that the strong criterion revealed very few clear winners (comparisons were done on a total of 224 motifs).
