# Supplementary material for: Functional Characterization of Transcription Factor Motifs Using Cross-species Comparison across Large Evolutionary Distances
Source: PLoS Comput Biol. 2010 Jan 29;6(1):e1000652. doi: 10.1371/journal.pcbi.1000652 (PMC2813253; doi:10.1371/journal.pcbi.1000652)
Supplement: Figure S3 — Performance of predicted motif - GO associations using cross-species comparison, evaluated based on genetics-based binding data. (0.27 MB DOC) [file pcbi.1000652.s003.doc]

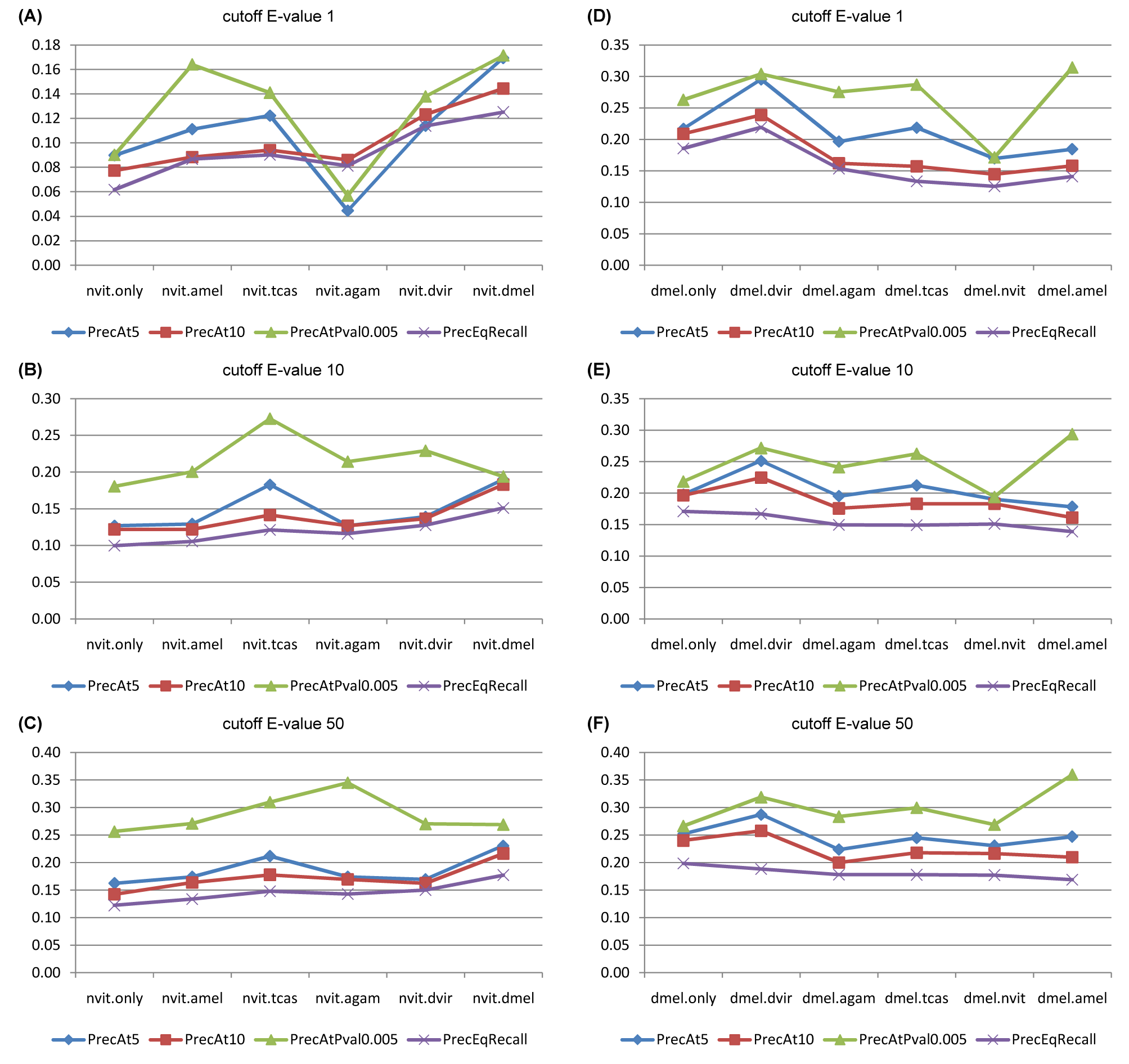


Figure S3. Performance of predicted motif – GO associations using cross-species comparison, evaluated based on genetics-based binding data. The prediction performance is shown as the precision of the top 5 and top 10 predictions per motif (“PrecAt5” and “PrecAt10”, respectively), the precision at a significance threshold (p-value) of 0.005 (“PrecAtPval0.005”), and as the point where precision equals recall (“PrecEqRecall”). Three different levels of significance (“cutoff E-value” 1 (A, D), 10 (B, E), and 50 (C, F)) were used to define the set of true associations, and the effect of cross-species comparison on the *Nasonia* (A-C) and *Drosophila* (D-F) motif function maps were reported separately.
