# Supplementary material for: Functional Characterization of Transcription Factor Motifs Using Cross-species Comparison across Large Evolutionary Distances
Source: PLoS Comput Biol. 2010 Jan 29;6(1):e1000652. doi: 10.1371/journal.pcbi.1000652 (PMC2813253; doi:10.1371/journal.pcbi.1000652)
Supplement: Figure S4 — Predicted motifs for Drosophila protein CG7056-PA and its ortholog in Nasonia using the online tool at http://ural.wustl.edu/flyhd. (0.17 MB DOC) [file pcbi.1000652.s004.doc]

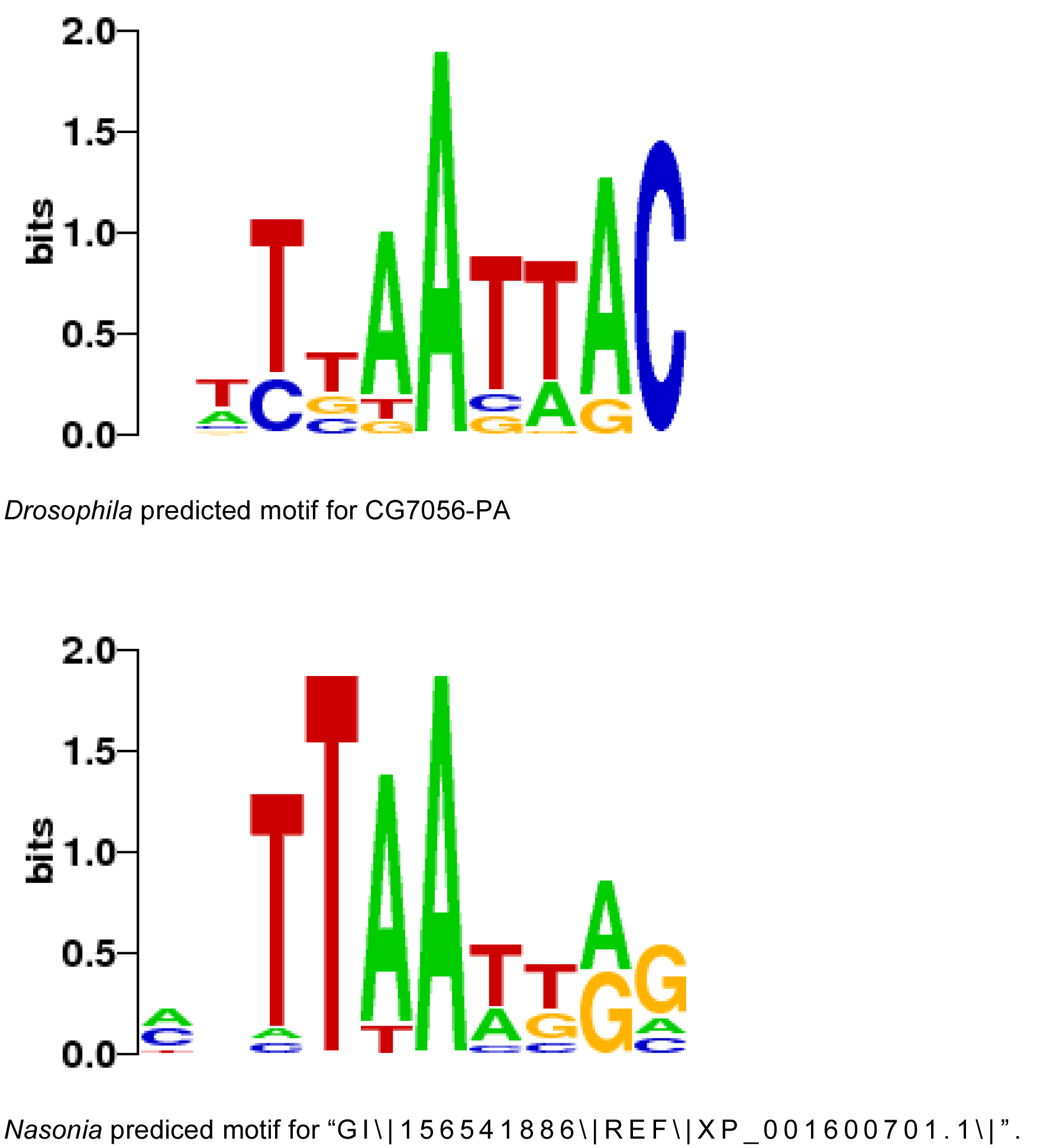


Figure S4. Predicted motifs for *Drosophila* protein CG7056-PA and its ortholog in *Nasonia* using the online tool at http://ural.wustl.edu/flyhd
