# Supplementary material for: Functional Characterization of Transcription Factor Motifs Using Cross-species Comparison across Large Evolutionary Distances
Source: PLoS Comput Biol. 2010 Jan 29;6(1):e1000652. doi: 10.1371/journal.pcbi.1000652 (PMC2813253; doi:10.1371/journal.pcbi.1000652)
Supplement: Table S1 — Correlation between motif characteristics and amenability to specific methods. (0.05 MB DOC) [file pcbi.1000652.s005.doc]

Table S1. Correlation between motif characteristics and amenability to specific methods.

| **Motif characteristic** | **Method** | **p-value^a^** | **#motifs** | **mean** | **median** |
| --- | --- | --- | --- | --- | --- |
| G/C content | site-LLR | **0.0031** | 62 | 0.35 | 0.32 |
|  | Stubb |  | 70 | 0.28 | 0.18 |
|  | SWAN | **0.0104** | 92 | 0.34 | 0.33 |
|  | Stubb |  | 70 | 0.28 | 0.18 |
|  | site-LLR | 0.6097 | 62 | 0.35 | 0.32 |
|  | SWAN |  | 92 | 0.34 | 0.33 |
| Length | site-LLR | 0.3399 | 62 | 8.9 | 8 |
|  | Stubb |  | 70 | 8.47 | 7 |
|  | SWAN | 0.3799 | 92 | 9.02 | 8 |
|  | Stubb |  | 70 | 8.47 | 7 |
|  | site-LLR | 0.9911 | 62 | 8.9 | 8 |
|  | SWAN |  | 92 | 9.02 | 8 |
| Information content^b^ | site-LLR | 0.5548 | 59 | 1.44 | 1.45 |
|  | Stubb |  | 69 | 1.42 | 1.44 |
|  | SWAN | 0.3062 | 86 | 1.39 | 1.43 |
|  | Stubb |  | 69 | 1.42 | 1.44 |
|  | site-LLR | 0.1416 | 59 | 1.44 | 1.45 |
|  | SWAN |  | 86 | 1.39 | 1.43 |

We compared each pair of methods (from site-LLR, Stubb and SWAN) by (i) collecting the sets of motifs on which each method was superior over the other, (ii) calculating motif characteristics (G/C content, length, or Information content) for each motif, and (iii) comparing these values between the motif sets associated with the pair of methods (using a two sample Wilcoxon rank sum test). We report as summary statistics for each motif set (i.e., the motifs on which one method was superior over the other), the number of motifs (**#motifs**), and mean and median values of a motif characteristic (**mean** and **median** respectively).

^a^p-value from two sample Wilcoxon rank sum test

^b^Average information content of motif positions whose information content ≥ 1
