# Supplementary material for: Functional Characterization of Transcription Factor Motifs Using Cross-species Comparison across Large Evolutionary Distances
Source: PLoS Comput Biol. 2010 Jan 29;6(1):e1000652. doi: 10.1371/journal.pcbi.1000652 (PMC2813253; doi:10.1371/journal.pcbi.1000652)
Supplement: Table S2 — Possible motif conservation scores and their semantics. See text for explanation of how. (0.03 MB DOC) [file pcbi.1000652.s006.doc]

Table S2. Possible motif conservation scores and their semantics. See text for explanation of how “key” residues were identified.

| ? | No domain found in *Drosophila* OR Strong structural template not found OR Different domains in *Drosophila* protein have their best matching domain in different genes in *Nasonia* |
| --- | --- |
| 1 | Domain found in *Drosophila* but strong ortholog not found in *Nasonia* |
| 2 | All domains in *Drosophila* protein have strong orthologous domains in the same *Nasonia* protein; at least one key residue changes to an amino acid in a different group. |
| 3 | No key residue undergoes between-group substitution, but at least one residue undergoes within-group substitution. |
| 4 | No key residue undergoes any substitution. |
