# Supplementary material for: Functional Characterization of Transcription Factor Motifs Using Cross-species Comparison across Large Evolutionary Distances
Source: PLoS Comput Biol. 2010 Jan 29;6(1):e1000652. doi: 10.1371/journal.pcbi.1000652 (PMC2813253; doi:10.1371/journal.pcbi.1000652)
Supplement: Table S3 — Motif functional associations common to Nasonia and Drosophila. (0.31 MB DOC) [file pcbi.1000652.s007.doc]

Table S3. Motif functional associations common to *Nasonia* and *Drosophila*. We tested for enrichment between a GO category and each motif, separately in *D. melanogaster* (D.mel p-value), *N. vitripennis* (N.vit p-value) and also computed a combined p-value from these two lineage-specific p-values. Q-values (for mutiple hypothesis correction) reported here were based on this combined p-value.

| **Num** | **Motif** | **MCS**^a^ | **Motif source** | **D.mel**  **p-value** | **N.vit**  **p-value** | **Combined**  **p-value** | **q-value** | **GO category** | **Explained**  **by**^b^ |
| --- | --- | --- | --- | --- | --- | --- | --- | --- | --- |
| 1 | I_SUH_01 | ? | T | 3.97E-06 | 0.0001 | 4.52E-09 | 1.01E-06 | Notch signaling pathway | - |
| 2 | I_GAGAFACTOR_Q6 | ? | T | 6.90E-09 | 0.0001 | 6.20E-09 | 9.99E-07 | regulation of transcription from RNA polymerase II promoter | - |
| 3 | hunchback | ? | T | 0.0001 | 0.0001 | 1.29E-08 | 1.04E-06 | regulation of transcription from RNA polymerase II promoter | - |
| 4 | bicoid.new.5 | 2 | B | 0.0001 | 0.0007 | 2.77E-07 | 0.0001 | posterior head segmentation | D(18) |
| 5 | Scr.new.7 | 4 | B | 0.0004 | 0.0006 | 4.57E-07 | 0.0001 | proteolysis | - |
| 6 | hunchback.new.5 | ? | B | 0.0002 | 0.0009 | 5.08E-07 | 0.0001 | nervous system development | - |
| 7 | hunchback.new.5 | ? | B | 0.0002 | 0.0012 | 1.07E-06 | 0.0002 | ectoderm development | D(6)  N(6) |
| 8 | bab1.txt | ? | F | 0.0007 | 0.0008 | 1.13E-06 | 0.0001 | regulation of transcription from RNA polymerase II promoter | - |
| 9 | CG18599.new.7 | 4 | B | 0.0005 | 0.0012 | 1.36E-06 | 0.0001 | proteolysis | - |
| 10 | Btn.new.7 | 3 | B | 0.0016 | 0.0002 | 1.68E-06 | 0.0001 | proteolysis | - |
| 11 | cad.txt | 4 | F | 0.0008 | 0.0013 | 2.21E-06 | 0.0004 | regulation of transcription, DNA-dependent | - |
| 12 | I_GAGAFACTOR_Q6 | ? | T | 0.0026 | 0.0001 | 3.88E-06 | 0.0009 | tracheal system development (sensu Insecta) | - |
| 13 | ftz.new.6 | 4 | B | 4.54E-05 | 0.0035 | 6.38E-06 | 0.0004 | proteolysis | - |
| 14 | bcd.txt | 2 | F | 0.0021 | 0.0015 | 6.46E-06 | 0.0007 | posterior head segmentation | N(4) |
| 15 | I_GAGAFACTOR_Q6 | ? | T | 0.0041 | 0.0002 | 9.11E-06 | 0.0020 | mesoderm development | D(111) |
| 16 | Hsf.txt | 2 | F | 4.08E-05 | 0.0045 | 1.01E-05 | 0.0023 | protein complex assembly | - |
| 17 | AbdB.new.7 | 4 | B | 0.0050 | 1.78E-05 | 1.24E-05 | 0.0010 | ectoderm development | - |
| 18 | bicoid.new.5 | 2 | B | 2.83E-05 | 0.0054 | 1.47E-05 | 0.0033 | trunk segmentation | - |
| 19 | Zen2.new.7 | 4 | B | 0.0003 | 0.0057 | 1.80E-05 | 0.0007 | proteolysis | N(5) |
| 20 | Antp.new.7 | 4 | B | 0.0001 | 0.0061 | 1.93E-05 | 0.0007 | proteolysis | N(10) |
| 21 | hunchback.new.4.10 | ? | B | 0.0034 | 0.0034 | 2.26E-05 | 0.0009 | regulation of transcription from RNA polymerase II promoter | D(3)  N(3) |
| 22 | I_GAGAFACTOR_Q6 | ? | T | 0.0066 | 0.0002 | 2.34E-05 | 0.0022 | regulation of transcription, DNA-dependent | - |
| 23 | I_GAGAFACTOR_Q6 | ? | T | 1.76E-06 | 0.0069 | 2.39E-05 | 0.0053 | dorsal closure | - |
| 24 | I_GAGAFACTOR_Q6 | ? | T | 0.0019 | 0.0055 | 2.73E-05 | 0.0061 | leg disc proximal/distal pattern formation | N(43) |
| 25 | I_MTTFA_01 | ? | T | 0.0058 | 0.0019 | 2.95E-05 | 0.0066 | apoptosis | - |
| 26 | zen.txt | 4 | F | 0.0069 | 0.0009 | 3.11E-05 | 0.0017 | ectoderm development | - |
| 27 | Hsf.txt | 2 | F | 8.50E-09 | 0.0086 | 3.69E-05 | 0.0083 | response to heat | - |
| 28 | Dfd.new.7 | 4 | B | 0.0007 | 0.0080 | 3.73E-05 | 0.0012 | proteolysis | N(10) |
| 29 | E5.new.7 | 3 | B | 0.0002 | 0.0085 | 3.82E-05 | 0.0035 | pattern specification | N(35) |
| 30 | CG12361.new.7 | 4 | B | 0.0057 | 0.0033 | 4.02E-05 | 0.0090 | cyclic nucleotide metabolism | - |
| 31 | Repo.new.7 | 4 | B | 0.0008 | 0.0083 | 4.14E-05 | 0.0082 | transmission of nerve impulse | N(169) |
| 32 | I_GAGAFACTOR_Q6 | ? | T | 0.0040 | 0.0053 | 4.33E-05 | 0.0097 | blastoderm segmentation | N(43) |
| 33 | Ems.new.7 | 3 | B | 0.0024 | 0.0069 | 4.37E-05 | 0.0035 | pattern specification | - |
| 34 | E5.new.7 | 3 | B | 0.0006 | 0.0088 | 4.48E-05 | 0.0089 | G-protein coupled receptor protein signaling pathway | - |
| 35 | Slou.new.7 | 4 | B | 0.0028 | 0.0069 | 4.70E-05 | 0.0035 | pattern specification | - |
| 53 | H20.new.7 | 3 | B | 0.0007 | 0.0102 | 0.0001 | 0.0025 | ectoderm development | - |
| 42 | Ftz.new.7 | 4 | B | 0.0109 | 0.0001 | 0.0001 | 0.0072 | antennal morphogenesis | - |
| 62 | I_ANTP_Q6 | 4 | T | 2.84E-06 | 0.0111 | 0.0001 | 0.0139 | central nervous system development | - |
| 41 | ftz.new.1 | 4 | B | 0.0112 | 0.0001 | 0.0001 | 0.0072 | antennal morphogenesis | - |
| 63 | hunchback.new.4.8 | ? | B | 0.0084 | 0.0030 | 0.0001 | 0.0145 | negative regulation of cardioblast cell fate specification | - |
| 59 | Ap.new.7 | 4 | B | 1.60E-05 | 0.0114 | 0.0001 | 0.0018 | proteolysis | N(10) |
| 67 | hb.txt | ? | F | 4.62E-05 | 0.0115 | 0.0001 | 0.0021 | regulation of transcription from RNA polymerase II promoter | N(130) |
| 66 | I_GAGAFACTOR_Q6 | ? | T | 0.0115 | 0.0001 | 0.0001 | 0.0152 | wing morphogenesis | D(52) |
| 51 | hunchback | ? | T | 0.0114 | 0.0005 | 0.0001 | 0.0158 | cell proliferation | D(3) |
| 56 | AbdB.new.7 | 4 | B | 0.0076 | 0.0045 | 0.0001 | 0.0165 | salivary gland development | D(118) |
| 36 | eve.new.6 | 4 | B | 0.0017 | 0.0104 | 0.0001 | 0.0123 | muscle development | - |
| 49 | Btn.new.7 | 3 | B | 0.0023 | 0.0107 | 0.0001 | 0.0188 | sensory perception of smell | - |
| 69 | AbdB.new.7 | 4 | B | 0.0069 | 0.0061 | 0.0001 | 0.0079 | gut mesoderm development | N(17) |
| 57 | Abd-B.txt | 4 | F | 0.0001 | 0.0131 | 0.0001 | 0.0028 | ectoderm development | - |
| 38 | I_GAGAFACTOR_Q6 | ? | T | 0.0027 | 0.0106 | 0.0001 | 0.0198 | imaginal disc growth | - |
| 45 | hb.txt | ? | F | 0.0013 | 0.0121 | 0.0001 | 0.0122 | periodic partitioning by pair rule gene | D(42) |
| 50 | I_GAGAFACTOR_Q6 | ? | T | 3.91E-08 | 0.0137 | 0.0001 | 0.0207 | eye development (sensu Endopterygota) | - |
| 70 | I_GAGAFACTOR_Q6 | ? | T | 3.16E-05 | 0.0140 | 0.0001 | 0.0223 | cell fate determination | - |
| 52 | Antp.txt | 4 | F | 0.0075 | 0.0069 | 0.0001 | 0.0079 | gut mesoderm development | - |
| 39 | CG4328.new.7 | 3 | B | 0.0074 | 0.0072 | 0.0001 | 0.0079 | gut mesoderm development | - |
| 37 | hunchback | ? | T | 0.0020 | 0.0127 | 0.0001 | 0.0122 | periodic partitioning by pair rule gene | D(3) |
| 43 | I_GAGAFACTOR_Q6 | ? | T | 2.30E-05 | 0.0147 | 0.0001 | 0.0105 | nervous system development | D(111) |
| 61 | I_GAGAFACTOR_Q6 | ? | T | 0.0065 | 0.0083 | 0.0001 | 0.0123 | muscle development | - |
| 48 | eve.new.6 | 4 | B | 0.0061 | 0.0091 | 0.0001 | 0.0089 | G-protein coupled receptor protein signaling pathway | N(34) |
| 54 | Abd-B.txt | 4 | F | 0.0064 | 0.0089 | 0.0001 | 0.0086 | posterior head segmentation | D(118)  N(4) |
| 55 | CG11617.new.7 | 2 | B | 0.0074 | 0.0080 | 0.0001 | 0.0262 | leg disc morphogenesis | - |
| 58 | Lbl.new.7 | 4 | B | 0.0069 | 0.0086 | 0.0001 | 0.0028 | proteolysis | N(10) |
| 46 | E5.new.7 | 3 | B | 4.96E-05 | 0.0156 | 0.0001 | 0.0028 | proteolysis | N(10) |
| 47 | I_GAGAFACTOR_Q6 | ? | T | 0.0133 | 0.0029 | 0.0001 | 0.0292 | regulation of cell proliferation | D(52) |
| 44 | ftz.txt | 4 | F | 0.0139 | 0.0023 | 0.0001 | 0.0296 | regulation of transcription | - |
| 40 | bcd.txt | 2 | F | 0.0112 | 0.0052 | 0.0001 | 0.0150 | blastoderm segmentation | N(14) |
| 60 | Ro.new.7 | 4 | B | 2.69E-05 | 0.0163 | 0.0001 | 0.0089 | G-protein coupled receptor protein signaling pathway | N(34) |
| 64 | I_GAGAFACTOR_Q6 | ? | T | 0.0022 | 0.0143 | 0.0001 | 0.0306 | branched duct epithelial cell fate determination (sensu Insecta) | - |
| 65 | hunchback.new.4.10 | ? | B | 0.0039 | 0.0129 | 0.0001 | 0.0105 | nervous system development | D(6)  N(6) |
| 68 | I_GAGAFACTOR_Q6 | ? | T | 0.0073 | 0.0100 | 0.0001 | 0.0334 | eye-antennal disc morphogenesis | D(52) |
| 91 | I_HAIRY_01 | 4 | T | 0.0002 | 0.0176 | 0.0002 | 0.0179 | central nervous system development | - |
| 75 | CG7056.new.7 | 2 | B | 0.0163 | 0.0020 | 0.0002 | 0.0045 | ectoderm development | - |
| 87 | I_GAGAFACTOR_Q6 | ? | T | 0.0129 | 0.0055 | 0.0002 | 0.0376 | tracheal epithelial cell migration (sensu Insecta) | D(68)  N(12) |
| 86 | bcd.txt | 2 | F | 0.0014 | 0.0171 | 0.0002 | 0.0168 | trunk segmentation | D(18)  N(14) |
| 76 | AbdB.new.7 | 4 | B | 0.0155 | 0.0029 | 0.0002 | 0.0046 | regulation of transcription from RNA polymerase II promoter | - |
| 83 | Zen2.new.7 | 4 | B | 0.0001 | 0.0187 | 0.0002 | 0.0089 | G-protein coupled receptor protein signaling pathway | N(34) |
| 72 | BH2.new.7 | 4 | B | 0.0089 | 0.0103 | 0.0002 | 0.0207 | eye development (sensu Endopterygota) | - |
| 90 | Trl.txt | ? | F | 0.0066 | 0.0127 | 0.0002 | 0.0416 | wing margin morphogenesis | - |
| 93 | Lbl.new.7 | 4 | B | 0.0001 | 0.0192 | 0.0002 | 0.0420 | phototransduction, visible light | - |
| 71 | ftz.new.6 | 4 | B | 0.0108 | 0.0087 | 0.0002 | 0.0424 | mesodermal cell fate specification | - |
| 73 | Lbl.new.7 | 4 | B | 0.0003 | 0.0192 | 0.0002 | 0.0157 | phototransduction, UV | D(79) |
| 77 | Lbl.new.7 | 4 | B | 0.0132 | 0.0065 | 0.0002 | 0.0328 | visual perception | - |
| 80 | odd.new.4 | ? | B | 0.0145 | 0.0055 | 0.0002 | 0.0112 | pattern specification | - |
| 82 | CG33980.new.7 | 2 | B | 0.0187 | 0.0013 | 0.0002 | 0.0242 | negative regulation of frizzled signaling pathway | N(143) |
| 79 | I_GAGAFACTOR_Q6 | ? | T | 0.0057 | 0.0143 | 0.0002 | 0.0448 | equator specification | D(111) |
| 81 | I_GAGAFACTOR_Q6 | ? | T | 0.0150 | 0.0051 | 0.0002 | 0.0450 | protein amino acid phosphorylation | D(23) |
| 88 | Hsf.txt | 2 | F | 3.98E-07 | 0.0202 | 0.0002 | 0.0460 | protein folding | - |
| 78 | ap.txt | 4 | F | 0.0003 | 0.0200 | 0.0002 | 0.0157 | phototransduction, UV | - |
| 74 | Rx.new.7 | 4 | B | 0.0194 | 0.0013 | 0.0002 | 0.0242 | negative regulation of frizzled signaling pathway | N(143) |
| 92 | CG12361.new.7 | 4 | B | 0.0052 | 0.0157 | 0.0002 | 0.0051 | ectoderm development | - |
| 85 | I_HAIRY_01 | 4 | T | 0.0122 | 0.0088 | 0.0002 | 0.0490 | embryonic development (sensu Insecta) | - |
| 84 | hunchback | ? | T | 0.0006 | 0.0206 | 0.0002 | 0.0168 | trunk segmentation | D(18)  N(3) |
| 89 | Oc.new.7 | 4 | B | 0.0143 | 0.0079 | 0.0002 | 0.0184 | central nervous system development | - |
| 102 | Pb.new.7 | ? | B | 0.0199 | 0.0026 | 0.0003 | 0.0052 | proteolysis | N(5) |
| 103 | caudal.new.1 | 4 | B | 0.0050 | 0.0177 | 0.0003 | 0.0134 | transmission of nerve impulse | D(138)  N(96) |
| 94 | caudal.new.4 | 4 | B | 0.0050 | 0.0177 | 0.0003 | 0.0134 | transmission of nerve impulse | D(138) |
| 108 | CG15696.new.7 | 2 | B | 0.0054 | 0.0174 | 0.0003 | 0.0157 | phototransduction, UV | - |
| 106 | AbdB.new.7 | 4 | B | 0.0032 | 0.0200 | 0.0003 | 0.0134 | transmission of nerve impulse | N(96) |
| 99 | Bcd.new.7 | 2 | B | 0.0089 | 0.0148 | 0.0003 | 0.0209 | tracheal system development (sensu Insecta) | - |
| 100 | bicoid.new.1 | 2 | B | 0.0089 | 0.0148 | 0.0003 | 0.0209 | tracheal system development (sensu Insecta) | - |
| 107 | NK71.new.7 | 4 | B | 0.0057 | 0.0179 | 0.0003 | 0.0157 | phototransduction, UV | - |
| 105 | I_TTK69_01 | ? | T | 0.0127 | 0.0112 | 0.0003 | 0.0058 | regulation of transcription from RNA polymerase II promoter | - |
| 104 | hunchback.new.5 | ? | B | 0.0048 | 0.0191 | 0.0003 | 0.0058 | regulation of transcription from RNA polymerase II promoter | D(42)  N(3) |
| 97 | Hgtx.new.7 | 4 | B | 0.0041 | 0.0201 | 0.0003 | 0.0328 | visual perception | - |
| 101 | Dl_11 | 2 | T | 0.0087 | 0.0156 | 0.0003 | 0.0329 | mesoderm development | - |
| 95 | Eve.new.7 | 4 | B | 0.0001 | 0.0250 | 0.0003 | 0.0059 | proteolysis | N(10) |
| 96 | AbdB.new.7 | 4 | B | 0.0196 | 0.0061 | 0.0003 | 0.0428 | proximal/distal pattern formation, imaginal disc | N(17) |
| 98 | Hgtx.new.7 | 4 | B | 0.0196 | 0.0065 | 0.0003 | 0.0059 | proteolysis | N(13) |
| 116 | abd-A.txt | 4 | F | 0.0220 | 0.0047 | 0.0004 | 0.0064 | ectoderm development | D(36) |
| 114 | hunchback.new.4.10 | ? | B | 0.0191 | 0.0078 | 0.0004 | 0.0403 | negative regulation of cardioblast cell fate specification | N(40) |
| 109 | I_GAGAFACTOR_Q6 | ? | T | 4.47E-07 | 0.0268 | 0.0004 | 0.0064 | ectoderm development | - |
| 111 | Abd-B.txt | 4 | F | 0.0013 | 0.0262 | 0.0004 | 0.0137 | transmission of nerve impulse | - |
| 117 | Dll.new.7 | 4 | B | 0.0207 | 0.0069 | 0.0004 | 0.0428 | proximal/distal pattern formation, imaginal disc | - |
| 118 | hunchback.new.4.10 | ? | B | 0.0240 | 0.0039 | 0.0004 | 0.0064 | ectoderm development | D(7)  N(7) |
| 113 | dl.txt | 2 | F | 0.0021 | 0.0259 | 0.0004 | 0.0295 | eye development (sensu Endopterygota) | - |
| 115 | Trl.txt | ? | F | 4.93E-07 | 0.0285 | 0.0004 | 0.0073 | regulation of transcription from RNA polymerase II promoter | D(2)  N(3) |
| 110 | bab1.txt | ? | F | 0.0178 | 0.0110 | 0.0004 | 0.0451 | brain development | - |
| 112 | Abd-B.txt | 4 | F | 3.51E-06 | 0.0286 | 0.0004 | 0.0293 | salivary gland development | - |
| 119 | Bsh.new.7 | 4 | B | 0.0025 | 0.0262 | 0.0004 | 0.0137 | transmission of nerve impulse | N(169) |
| 125 | grh.txt | ? | F | 0.0003 | 0.0297 | 0.0005 | 0.0432 | regulation of transcription | - |
| 126 | CG32105.new.7 | 4 | B | 0.0229 | 0.0075 | 0.0005 | 0.0451 | brain development | - |
| 123 | PdhP.new.7 | 4 | B | 0.0304 | 0.0003 | 0.0005 | 0.0070 | ectoderm development | - |
| 124 | Ftz.new.7 | 4 | B | 0.0240 | 0.0070 | 0.0005 | 0.0187 | pattern specification | N(35) |
| 122 | Inv.new.7 | 4 | B | 0.0089 | 0.0228 | 0.0005 | 0.0187 | pattern specification | N(83) |
| 121 | Oc.new.7 | 4 | B | 0.0042 | 0.0282 | 0.0005 | 0.0324 | regulation of transcription, DNA-dependent | - |
| 120 | tailless.new.1 | 2 | B | 0.0087 | 0.0240 | 0.0005 | 0.0086 | proteolysis | - |
| 127 | CG32105.new.7 | 4 | B | 0.0335 | 0.0003 | 0.0006 | 0.0074 | ectoderm development | - |
| 130 | dl.txt | 2 | F | 0.0001 | 0.0337 | 0.0006 | 0.0432 | regulation of transcription | N(65) |
| 132 | abd-A.txt | 4 | F | 0.0018 | 0.0322 | 0.0006 | 0.0293 | salivary gland development | - |
| 129 | hb.txt | ? | F | 0.0330 | 0.0010 | 0.0006 | 0.0074 | ectoderm development | D(111) |
| 131 | CG4328.new.7 | 3 | B | 0.0018 | 0.0332 | 0.0006 | 0.0293 | salivary gland development | - |
| 128 | hb.txt | ? | F | 0.0309 | 0.0050 | 0.0006 | 0.0291 | nervous system development | D(57)  N(130) |
| 135 | hunchback | ? | T | 0.0003 | 0.0356 | 0.0007 | 0.0291 | nervous system development | N(3,6) |
| 139 | exd.txt | 4 | F | 0.0348 | 0.0012 | 0.0007 | 0.0293 | salivary gland development | D(118) |
| 133 | en.txt | 4 | F | 0.0268 | 0.0096 | 0.0007 | 0.0211 | pattern specification | N(35) |
| 136 | I_GAGAFACTOR_Q6 | ? | T | 0.0020 | 0.0343 | 0.0007 | 0.0451 | brain development | - |
| 134 | I_GAGAFACTOR_Q6 | ? | T | 0.0082 | 0.0291 | 0.0007 | 0.0423 | hindgut morphogenesis | D(2) |
| 137 | caudal.new.5 | 4 | B | 0.0001 | 0.0371 | 0.0007 | 0.0198 | transmission of nerve impulse | N(169) |
| 140 | exd.txt | 4 | F | 0.0366 | 0.0009 | 0.0007 | 0.0083 | ectoderm development | D(111) |
| 138 | Oc.new.7 | 4 | B | 0.0031 | 0.0350 | 0.0007 | 0.0423 | hindgut morphogenesis | D(152) |
| 142 | ftz.new.1 | 4 | B | 0.0002 | 0.0383 | 0.0008 | 0.0261 | G-protein coupled receptor protein signaling pathway | N(34) |
| 141 | I_MTTFA_01 | ? | T | 0.0042 | 0.0345 | 0.0008 | 0.0113 | proteolysis | - |
| 144 | CG32105.new.7 | 4 | B | 0.0383 | 0.0004 | 0.0008 | 0.0295 | negative regulation of frizzled signaling pathway | N(127) |
| 146 | en.txt | 4 | F | 0.0266 | 0.0137 | 0.0008 | 0.0451 | brain development | - |
| 145 | hunchback | ? | T | 0.0104 | 0.0299 | 0.0008 | 0.0454 | posterior head segmentation | D(92)  N(3,4) |
| 143 | AbdB.new.7 | 4 | B | 0.0263 | 0.0145 | 0.0008 | 0.0434 | tracheal system development (sensu Insecta) | N(17) |
| 150 | Bcd.new.7 | 2 | B | 0.0030 | 0.0383 | 0.0009 | 0.0423 | hindgut morphogenesis | D(152) |
| 147 | bicoid.new.1 | 2 | B | 0.0030 | 0.0383 | 0.0009 | 0.0423 | hindgut morphogenesis | D(152) |
| 148 | Inv.new.7 | 4 | B | 0.0189 | 0.0228 | 0.0009 | 0.0295 | negative regulation of frizzled signaling pathway | N(143) |
| 152 | Pb.new.7 | ? | B | 3.01E-05 | 0.0426 | 0.0009 | 0.0261 | G-protein coupled receptor protein signaling pathway | N(34) |
| 151 | Awh.new.7 | 4 | B | 0.0374 | 0.0057 | 0.0009 | 0.0295 | negative regulation of frizzled signaling pathway | N(143) |
| 153 | Ptx1.new.7 | 4 | B | 0.0026 | 0.0407 | 0.0009 | 0.0423 | hindgut morphogenesis | - |
| 149 | CG32532.new.7 | 3 | B | 0.0375 | 0.0059 | 0.0009 | 0.0295 | negative regulation of frizzled signaling pathway | N(143) |
| 155 | Repo.new.7 | 4 | B | 0.0381 | 0.0059 | 0.0010 | 0.0295 | negative regulation of frizzled signaling pathway | N(143) |
| 156 | odd.new.1 | ? | B | 0.0281 | 0.0161 | 0.0010 | 0.0099 | ectoderm development | D(156) |
| 157 | odd.new.6 | ? | B | 0.0281 | 0.0161 | 0.0010 | 0.0099 | ectoderm development | - |
| 154 | I_E74A_01 | 4 | T | 0.0017 | 0.0426 | 0.0010 | 0.0434 | tracheal system development (sensu Insecta) | - |
| 159 | Slou.new.7 | 4 | B | 0.0391 | 0.0069 | 0.0011 | 0.0295 | negative regulation of frizzled signaling pathway | N(143) |
| 160 | Zen.new.7 | 4 | B | 0.0002 | 0.0464 | 0.0011 | 0.0261 | G-protein coupled receptor protein signaling pathway | N(34) |
| 161 | AbdB.new.7 | 4 | B | 0.0297 | 0.0175 | 0.0011 | 0.0337 | nervous system development | N(17) |
| 158 | Oc.new.7 | 4 | B | 0.0410 | 0.0068 | 0.0011 | 0.0279 | transmission of nerve impulse | - |
| 163 | Abd-B.txt | 4 | F | 0.0013 | 0.0466 | 0.0012 | 0.0434 | tracheal system development (sensu Insecta) | - |
| 164 | Al.new.7 | 4 | B | 0.0182 | 0.0303 | 0.0012 | 0.0261 | G-protein coupled receptor protein signaling pathway | N(34) |
| 168 | Dll.new.7 | 4 | B | 1.18E-05 | 0.0482 | 0.0012 | 0.0261 | G-protein coupled receptor protein signaling pathway | N(34) |
| 166 | NK71.new.7 | 4 | B | 0.0403 | 0.0083 | 0.0012 | 0.0295 | negative regulation of frizzled signaling pathway | N(143) |
| 167 | opa.new.1 | 4 | B | 0.0068 | 0.0421 | 0.0012 | 0.0337 | nervous system development | - |
| 162 | opa.new.6 | 4 | B | 0.0068 | 0.0421 | 0.0012 | 0.0337 | nervous system development | - |
| 165 | Antp.new.7 | 4 | B | 0.0490 | 8.31E-06 | 0.0012 | 0.0115 | ectoderm development | - |
| 170 | Awh.new.7 | 4 | B | 0.0496 | 0.0004 | 0.0013 | 0.0279 | transmission of nerve impulse | - |
| 169 | bab1.txt | ? | F | 0.0202 | 0.0304 | 0.0013 | 0.0115 | ectoderm development | D(8) |
| 171 | CG32532.new.7 | 3 | B | 0.0408 | 0.0196 | 0.0018 | 0.0254 | proteolysis | N(10) |
| 172 | dsx | ? | L | 0.0304 | 0.0324 | 0.0020 | 0.0353 | G-protein coupled receptor protein signaling pathway | - |
| 173 | caudal.new.5 | 4 | B | 0.0273 | 0.0380 | 0.0021 | 0.0161 | ectoderm development | D(49) |
| 174 | Scr.new.7 | 4 | B | 0.0395 | 0.0317 | 0.0025 | 0.0178 | ectoderm development | - |
| 175 | Odsh.new.7 | ? | B | 0.0441 | 0.0291 | 0.0027 | 0.0180 | ectoderm development | - |
| 176 | I_HAIRY_01 | 4 | T | 0.0275 | 0.0476 | 0.0028 | 0.0182 | ectoderm development | - |
| 177 | I_SUH_01 | ? | T | 0.0364 | 0.0499 | 0.0037 | 0.0200 | ectoderm development | D(1)  N(1) |

Motif source: B, B1H; F, flyreg.org data; T, Transfac; L, literature

^a^Motif conservation score

^b^Associations statistically explaining the association in the current row at a cutoff p-value 0.05. Associations are represented by row numbers (column 1) and the redundancy test was done separately for *Drosophila* (“D”) and *Nasonia* (“N”).
