# Supplementary material for: Functional Characterization of Transcription Factor Motifs Using Cross-species Comparison across Large Evolutionary Distances
Source: PLoS Comput Biol. 2010 Jan 29;6(1):e1000652. doi: 10.1371/journal.pcbi.1000652 (PMC2813253; doi:10.1371/journal.pcbi.1000652)
Supplement: Table S5 — Motif associations with genes up-regulated in response to Manganese treatment in honeybees. (0.11 MB DOC) [file pcbi.1000652.s009.doc]

Table S5. Motif associations with genes up-regulated in response to Manganese treatment in honeybees. P-values of association with the gene set defined in *Apis* (A.mel) as well their orthologous gene sets in *Nasonia* (N.vit) and *Drosophila* (D.mel) are shown in the last three columns. Only associations with p-value ≤ 0.01 in *Apis* are shown. Of 67 such associations, 27 pass the “evolutionary filter” of having p-value ≥ 0.10 in *Nasonia* as well as in *Drosophila*.

| **Gene set** | **Motif** | **MCS**^a^ | **Motif source** | **A.mel** | **p-value**  **N.vit** | **D.mel** |
| --- | --- | --- | --- | --- | --- | --- |
| MnHigh | Deaf1.txt | ? | F | **4.2E-07^*^** | 0.178 | 0.213 |
| MnHigh | BH1.new.7 | 4 | B | **9.1E-07^*^** | 0.228 | **0.031^**^** |
| MnHigh | kruppel.new.4 | 2 | B | **1.9E-06^*^** | **0.041^**^** | 0.591 |
| MnHigh | Hsf.txt | 2 | F | **8.9E-06^*^** | **0.026^**^** | **0.048^**^** |
| MnHigh | Antp.txt | 4 | F | **1.5E-05^*^** | **0.006^*^** | 0.405 |
| MnHigh | kruppel.new.2 | 2 | B | **3.2E-05^*^** | **0.095^***^** | 0.201 |
| MnHigh | dl.txt | 2 | F | **3.6E-05^*^** | **0.023^**^** | **0.009^*^** |
| MnHigh | Ftz.new.7 | 4 | B | **5.1E-05^*^** | **0.068^***^** | 0.511 |
| MnLow | byn.txt | 4 | F | **5.4E-05^*^** | 0.445 | 0.996 |
| MnHigh | C15.new.7 | 3 | B | **5.5E-05^*^** | **0.003^*^** | 0.272 |
| MnHigh | I_GAGAFACTOR_Q6 | ? | T | **7.8E-05^*^** | **1.3E-06^*^** | **0.055^***^** |
| MnLow | gt.txt | 3 | F | **8.0E-05^*^** | 0.706 | 0.937 |
| MnHigh | Dfd.txt | 4 | F | **9.6E-05^*^** | **0.055^***^** | 0.307 |
| MnHigh | I_DFD_01 | 4 | T | **1.5E-04^*^** | **0.009^*^** | **0.076^***^** |
| MnHigh | I_DRI_01 | 4 | T | **1.6E-04^*^** | **0.035^**^** | 0.123 |
| MnHigh | Ubx.txt | 4 | F | **1.8E-04^*^** | **0.058^***^** | 0.162 |
| MnHigh | PdhP.new.7 | 4 | B | **1.8E-04^*^** | 0.114 | **0.069^***^** |
| MnHigh | Dll.new.7 | 4 | B | **2.0E-04^*^** | **0.025^**^** | 0.455 |
| MnHigh | Unc4.new.7 | 3 | B | **3.0E-04^*^** | 0.101 | 0.237 |
| MnHigh | Adf1.txt | ? | F | **3.3E-04^*^** | **0.023^**^** | **0.034^**^** |
| MnLow | slbo.txt | 4 | F | **3.9E-04^*^** | 0.613 | 0.834 |
| MnHigh | CG11085.new.7 | ? | B | **4.4E-04^*^** | **0.027^**^** | **0.037^**^** |
| MnHigh | Bsh.new.7 | 4 | B | **4.8E-04^*^** | **0.030^**^** | 0.610 |
| MnHigh | Abd-A.new.7 | 4 | B | **0.001^*^** | **0.072^***^** | 0.697 |
| MnHigh | En.new.7 | 4 | B | **0.001^*^** | 0.294 | 0.499 |
| MnHigh | Hmx.new.7 | 4 | B | **0.001^*^** | **0.003^*^** | 0.279 |
| MnHigh | Dstat | ? | T | **0.001^*^** | **7.5E-05^*^** | 0.279 |
| MnHigh | CG32532.new.7 | 3 | B | **0.001^*^** | **0.095^***^** | 0.237 |
| MnHigh | CG32105.new.7 | 4 | B | **0.001^*^** | 0.218 | **0.069^***^** |
| MnHigh | BH2.new.7 | 4 | B | **0.001^*^** | 0.154 | **0.057^***^** |
| MnHigh | Kr.txt | 2 | F | **0.001^*^** | 0.860 | 0.724 |
| MnHigh | I_ADF1_Q6 | ? | T | **0.001^*^** | **0.068^***^** | **0.007^*^** |
| MnHigh | bin.txt | 2 | F | **0.001^*^** | 0.225 | **0.024^**^** |
| MnHigh | pan.txt | 4 | F | **0.001^*^** | 0.721 | 0.144 |
| MnHigh | kni.txt | 4 | F | **0.001^*^** | **0.007^*^** | 0.356 |
| MnHigh | CG11294.new.7 | 4 | B | **0.001^*^** | 0.351 | 0.905 |
| MnHigh | Trl.txt | ? | F | **0.001^*^** | **5.8E-07^*^** | **0.072^***^** |
| MnHigh | ftz.new.1 | 4 | B | **0.001^*^** | **0.055^***^** | 0.610 |
| MnHigh | NK71.new.7 | 4 | B | **0.002^*^** | 0.136 | **0.069^***^** |
| MnHigh | prd.new.6 | 2 | B | **0.002^*^** | 0.415 | 0.279 |
| MnHigh | Slou.new.7 | 4 | B | **0.002^*^** | **0.015^**^** | 0.105 |
| MnHigh | z.txt | ? | F | **0.002^*^** | 0.200 | 0.761 |
| MnLow | Exd.new.7 | 4 | B | **0.002^*^** | **0.002^*^** | 0.970 |
| MnHigh | Tup.new.7 | 4 | B | **0.002^*^** | **0.009^*^** | **0.032^**^** |
| MnHigh | abd-A.txt | 4 | F | **0.002^*^** | 0.600 | **7.9E-05^*^** |
| MnHigh | opa.new.6 | 4 | B | **0.003^*^** | 0.556 | **0.095^***^** |
| MnHigh | opa.new.1 | 4 | B | **0.003^*^** | 0.556 | **0.095^***^** |
| MnHigh | Hbn.new.7 | 2 | B | **0.003^*^** | 0.447 | 0.173 |
| MnHigh | Ubx.new.7 | 4 | B | **0.003^*^** | 0.266 | 0.184 |
| MnHigh | prd-all.new.1 | 2 | B | **0.003^*^** | 0.760 | 0.445 |
| MnHigh | Mad.txt | 4 | F | **0.003^*^** | 0.766 | 0.168 |
| MnHigh | Lim1.new.7 | ? | B | **0.004^*^** | 0.600 | 0.647 |
| MnHigh | Dl_11 | 2 | T | **0.004^*^** | 0.474 | 0.279 |
| MnHigh | Antp.new.7 | 4 | B | **0.004^*^** | 0.447 | 0.571 |
| MnLow | I_ZEN_Q6 | 4 | T | **0.004^*^** | 0.929 | 0.425 |
| MnHigh | Pph13.new.7 | 4 | B | **0.004^*^** | 0.439 | 0.269 |
| MnHigh | Lab.new.7 | 4 | B | **0.004^*^** | 0.136 | 0.303 |
| MnHigh | Repo.new.7 | 4 | B | **0.005^*^** | 0.562 | 0.355 |
| MnHigh | I_SUH_01 | ? | T | **0.005^*^** | 0.871 | 0.357 |
| MnHigh | I_CROC_01 | 4 | T | **0.005^*^** | 0.379 | 0.355 |
| MnHigh | CG15696.new.7 | 2 | B | **0.005^*^** | 0.558 | 0.228 |
| MnHigh | giant.new.4 | 3 | B | **0.006^*^** | 0.170 | 0.792 |
| MnHigh | CG4136.new.7 | 2 | B | **0.006^*^** | 0.202 | 0.737 |
| MnHigh | br-Z4.txt | ? | F | **0.007^*^** | **0.026^**^** | 0.617 |
| MnHigh | en.txt | 4 | F | **0.008^*^** | **0.005^*^** | 0.455 |
| MnHigh | Dref.txt | ? | F | **0.008^*^** | **0.040^**^** | 0.932 |
| MnHigh | Odsh.new.7 | ? | B | **0.009^*^** | 0.447 | 0.237 |
| MnHigh | CG13424.new.7 | 4 | B | **0.009^*^** | 0.187 | 0.168 |
| MnHigh | btd.new.6 | ? | B | **0.010^*^** | 0.883 | 0.112 |
| MnHigh | H20.new.7 | 3 | B | **0.010^*^** | **0.003^*^** | 0.132 |
| MnHigh | Lbl.new.7 | 4 | B | **0.010^*^** | **0.063^***^** | 0.571 |
| MnHigh | I_DREF_Q3 | ? | T | **0.010^*^** | 0.159 | 0.999 |

^*^: p-value < 0.01

^**^: 0.01 ≤ p-value < 0.05

^***^: 0.05 ≤ p-value < 0.1

^a^Motif conservation score

Motif source: B, B1H; F, flyreg.org data; T, Transfac
