# Supplementary material for: Functional Characterization of Transcription Factor Motifs Using Cross-species Comparison across Large Evolutionary Distances
Source: PLoS Comput Biol. 2010 Jan 29;6(1):e1000652. doi: 10.1371/journal.pcbi.1000652 (PMC2813253; doi:10.1371/journal.pcbi.1000652)
Supplement: Table S6 — Enrichment p-values for the oxidative phosphorylation gene set in Nasonia. (0.05 MB DOC) [file pcbi.1000652.s010.doc]

Table S6A. Enrichment p-values for the oxidative phosphorylation gene set in *Nasonia* with all (224) motifs in compendium. Only associations with p-value < 0.05 (without correction) are shown.

| **Motif** | **MCS**^a^ | **Motif**  **source** | **p-value** | **q-value** | **#intersection^b^** | **#motif targets^c^** | **#genes in gene set^d^** | **#total^e^** |
| --- | --- | --- | --- | --- | --- | --- | --- | --- |
| btd.new.6 | ? | B | 0.0117 | 1 | 10 | 696 | 58 | 9097 |
| V_AP1_C | 4 | T | 0.0321 | 1 | 9 | 701 | 58 | 9097 |
| pho.txt | 4 | F | 0.0362 | 1 | 9 | 716 | 58 | 9097 |
| PR1 | ? | L | 0.0461 | 1 | 9 | 749 | 58 | 9097 |
| I_ABDB_01 | 4 | T | 0.0465 | 1 | 9 | 750 | 58 | 9097 |

Motif source: B, B1H; F, flyreg.org data; T, Transfac; L, literature

^a^Motif conservation score

^b^Number of genes common in motif targets and oxidative phosphorylation gene set

^c^Number of motif target genes

^d^Number of genes in oxidative phosphorylation gene set

^e^Total number of genes in the analysis

Table S6B. Enrichment p-values for the oxidative phosphorylation gene set “Complex 1” in *Nasonia* with all motifs.

| **Motif** | **MCS**^a^ | **Motif**  **source** | **p-value** | **q-value** | **#intersection^b^** | **#motif targets^c^** | **#genes in gene set^d^** | **#total^e^** |
| --- | --- | --- | --- | --- | --- | --- | --- | --- |
| pho.txt | 4 | F | 0.0165 | 1 | 6 | 716 | 27 | 9097 |
| kruppel | 2 | T | 0.0200 | 1 | 6 | 747 | 27 | 9097 |
| run.new.6 | 4 | B | 0.0498 | 1 | 5 | 691 | 27 | 9097 |
| runt.new.1 | 4 | B | 0.0498 | 1 | 5 | 691 | 27 | 9097 |

Motif source: B, B1H; F, flyreg.org data; T, Transfac

^a^Motif conservation score

^b^Number of genes common in motif targets and oxidative phosphorylation gene set

^c^Number of motif target genes

^d^Number of genes in oxidative phosphorylation gene set

^e^Total number of genes in the analysis

Table S6C. Enrichment p-values for the oxidative phosphorylation gene set “Complex 2” in *Nasonia* with all motifs.

| **Motif** | **MCS**^a^ | **Motif**  **source** | **p-value** | **q-value** | **#intersection^b^** | **#motif targets^c^** | **#genes in gene set^d^** | **#total^e^** |
| --- | --- | --- | --- | --- | --- | --- | --- | --- |
| btd.new.6 | ? | B | 0.0017 | 0.3768 | 3 | 696 | 4 | 9097 |
| I_TTK69_01 | ? | T | 0.0319 | 1.0000 | 2 | 699 | 4 | 9097 |

Motif source: B, B1H

^a^Motif conservation score

^b^Number of genes common in motif targets and oxidative phosphorylation gene set

^c^Number of motif target genes

^d^Number of genes in oxidative phosphorylation gene set

^e^Total number of genes in the analysis

Table S6D. Enrichment p-values for the oxidative phosphorylation gene set “Complex 3” in *Nasonia* with all motifs.

| **Motif** | **MCS**^a^ | **Motif**  **source** | **p-value** | **q-value** | **#intersection^b^** | **#motif targets^c^** | **#genes in gene set^d^** | **#total^e^** |
| --- | --- | --- | --- | --- | --- | --- | --- | --- |
| giant.new.1 | 3 | B | 0.0025 | 0.4574 | 4 | 652 | 9 | 9097 |
| dichaete.new.5 | 4 | B | 0.0041 | 0.4574 | 4 | 748 | 9 | 9097 |
| giant.new.4 | 3 | B | 0.0198 | 1.0000 | 3 | 625 | 9 | 9097 |
| dl.txt | 2 | F | 0.0301 | 1.0000 | 3 | 731 | 9 | 9097 |

Motif source: B, B1H; F, flyreg.org data

^a^Motif conservation score

^b^Number of genes common in motif targets and oxidative phosphorylation gene set

^c^Number of motif target genes

^d^Number of genes in oxidative phosphorylation gene set

^e^Total number of genes in the analysis
