# Supplementary material for: Functional Characterization of Transcription Factor Motifs Using Cross-species Comparison across Large Evolutionary Distances
Source: PLoS Comput Biol. 2010 Jan 29;6(1):e1000652. doi: 10.1371/journal.pcbi.1000652 (PMC2813253; doi:10.1371/journal.pcbi.1000652)
Supplement: Text S1 — Chemoreceptor genes in Nasonia. (0.20 MB DOC) [file pcbi.1000652.s011.doc]

*Chemoreceptor genes in Nasonia*

Here, we illustrate an analysis where motif associations are compared not only across species, but also across different function categories that may be biologically and evolutionarily related. Chemoperception is a primary sense of insects, and is involved for example in mate recognition, and identification and functional understanding of chemoreceptors is critical in the study of this aspect of insect biology. Olfactory receptor (ORs) and gustatory receptor (GRs) genes have been annotated previously in *Drosophila* [1] and more recently in *Nasonia* [2], where 220 OR and 47 GR genes were identified. Here, we performed a systematic study of the *cis*-regulatory regions of these gene classes in order to identify the likely transcriptional regulators of chemoreceptor activity (Table S7). We found the OR gene set in *Nasonia* to be significantly associated with the motif of TTK (p-value 4E-7; Table S7A), a factor that was previously identified as regulating olfactory map development in *D.mel.* [3]. Highly significant associations were also observed for the KR (p-value 4E-5), OVO (p-value 6E-5) and RUN (p-value 0.003) motifs. Interestingly, the RUN motif is very similar to that of the TF LZ, which is known to regulate OR genes [4]. Analysis of the OR gene set in *D.mel* revealed that the association with the TTK motif is conserved (p-value 0.01); however, the strongest associations here were for the TTAATTG motif that corresponds to several homeodomain factors, including TUP, B-H2 and ABD-A (p-value < 1E-8) (Table S7C). Comparison of the enrichment analysis across species and in ORs as well as GRs revealed interesting commonalities in the predicted regulatory associations in these four cases (Table S8). Three motifs had associations present in both species (TTK for ORs, SNA and CROC for GRs), while three were associated with both ORs and GRs in *Nasonia* (SNA, PR1, and EXD).

Table S7A. Enrichment p-values for olfactory receptor genes in *Nasonia*

| **Motif** | **MCS**^a^ | **Motif**  **source** | **Motif best site** | **p-value** | **q-value** | **#common^b^** | **#motif targets^c^** | **#genes in gene set^d^** | **#total^e^** |
| --- | --- | --- | --- | --- | --- | --- | --- | --- | --- |
| ttk.txt | ? | F | GCCAGGACCTTG | 4.72E-07 | 0.0001 | 39 | 711 | 220 | 9342 |
| kruppel.new.4 | 2 | B | CAAAGGGTTA | 4.44E-05 | 0.0042 | 33 | 675 | 220 | 9342 |
| I_OVO_01 | ? | T | AGTAACAGT | 0.0001 | 0.0042 | 35 | 744 | 220 | 9342 |
| run.new.6 | 4 | B | TTGCGGTTA | 0.0025 | 0.0900 | 28 | 679 | 220 | 9342 |
| I_SN_01 | ? | T | GACAGGTGCA | 0.0028 | 0.0900 | 28 | 684 | 220 | 9342 |
| PR1 | ? | L | CCAAT | 0.0033 | 0.0920 | 30 | 758 | 220 | 9342 |
| Exd.new.7 | 4 | B | TGACAA | 0.0052 | 0.1285 | 30 | 782 | 220 | 9342 |
| I_dTCF_1 | 4 | T | CCTTTGATCTT | 0.0173 | 0.3330 | 21 | 544 | 220 | 9342 |
| odd.new.1 | ? | B | AACAGTAGCAG | 0.0272 | 0.4357 | 23 | 639 | 220 | 9342 |
| pho.txt | 4 | F | GCNGGTTATGGCTNC | 0.0343 | 0.5117 | 25 | 725 | 220 | 9342 |
| ct.new.7 | 4 | B | TTGAAC | 0.0404 | 0.5662 | 26 | 773 | 220 | 9342 |

Motif source: B, B1H; F, flyreg.org data; T, Transfac; L, literature

^a^Motif conservation score

^b^Number of genes common in motif targets and genes in the gene set

^c^Number of motif target genes

^d^Number of genes in the gene set

^e^Total number of genes in the analysis

Table S7B. Enrichment p-values for gustatory receptor genes in *Nasonia*

| **Motif** | **MCS**^a^ | **Motif**  **source** | **Motif best site** | **p-value** | **q-value** | **#common^b^** | **#motif targets^c^** | **#genes in gene set^d^** | **#total^e^** |
| --- | --- | --- | --- | --- | --- | --- | --- | --- | --- |
| Dref.txt | ? | F | TNTTATCGATAA | 0.0011 | 0.2010 | 11 | 47 | 47 | 9342 |
| I_SN_01 | ? | T | GACAGGTGCA | 0.0018 | 0.2010 | 10 | 47 | 47 | 9342 |
| Exd.new.7 | 4 | B | TGACAA | 0.0048 | 0.3221 | 10 | 47 | 47 | 9342 |
| Optix.new.7 | 4 | B | TGATA | 0.0058 | 0.3221 | 10 | 47 | 47 | 9342 |
| PR1 | ? | L | CCAAT | 0.0120 | 0.5190 | 9 | 47 | 47 | 9342 |
| slbo.txt | 4 | F | ATTGCAAA | 0.0139 | 0.5190 | 9 | 47 | 47 | 9342 |
| Lbl.new.7 | 4 | B | TAATTA | 0.0232 | 0.6890 | 8 | 47 | 47 | 9342 |
| Dfd.txt | 4 | F | TTAATTAT | 0.0246 | 0.6890 | 8 | 47 | 47 | 9342 |
| onecut.new.7 | 4 | B | TTGATTT | 0.0319 | 0.7930 | 8 | 47 | 47 | 9342 |
| Deaf1.txt | ? | F | TTCGT | 0.0405 | 0.9009 | 8 | 47 | 47 | 9342 |
| I_CROC_01 | 4 | T | AANAATAAATAT | 0.0442 | 0.9009 | 8 | 47 | 47 | 9342 |

Motif source: B, B1H; F, flyreg.org data; T, Transfac

^a^Motif conservation score

^b^Number of genes common in motif targets and genes in the gene set

^c^Number of motif target genes

^d^Number of genes in the gene set

^e^Total number of genes in the analysis

Table S7C. Enrichment p-values for olfactory receptor genes in *Drosophila*

| **Motif** | **MCS**^a^ | **Motif**  **source** | **Motif best site** | **p-value** | **q-value** | **#common^b^** | **#motif targets^c^** | **#genes in gene set^d^** | **#total^e^** |
| --- | --- | --- | --- | --- | --- | --- | --- | --- | --- |
| Tup.new.7 | 4 | B | TTAATTG | 1.30E-09 | 1.90E-07 | 22 | 1182 | 60 | 13987 |
| BH2.new.7 | 4 | B | TTAATTG | 1.70E-09 | 1.90E-07 | 22 | 1199 | 60 | 13987 |
| abd-A.txt | 4 | F | TTAATTGA | 6.90E-09 | 5.15E-07 | 21 | 1173 | 60 | 13987 |
| NK71.new.7 | 4 | B | TTAATTG | 1.12E-08 | 6.27E-07 | 21 | 1205 | 60 | 13987 |
| Abd-B.txt | 4 | B | TTTTATGA | 9.94E-08 | 4.45E-06 | 19 | 1114 | 60 | 13987 |
| I_EN_Q6 | 4 | T | TAATTGG | 2.42E-07 | 6.09E-06 | 19 | 1179 | 60 | 13987 |
| Hbn.new.7 | 2 | B | TTAATTA | 2.52E-07 | 6.09E-06 | 19 | 1182 | 60 | 13987 |
| Inv.new.7 | 4 | B | CTAATTA | 2.80E-07 | 6.09E-06 | 19 | 1190 | 60 | 13987 |
| CG13424.new.7 | 4 | B | TTAATTG | 2.91E-07 | 6.09E-06 | 19 | 1193 | 60 | 13987 |
| Unc4.new.7 | 3 | B | TTAATTG | 2.91E-07 | 6.09E-06 | 19 | 1193 | 60 | 13987 |
| CG32532.new.7 | 3 | B | TTAATTA | 2.99E-07 | 6.09E-06 | 19 | 1195 | 60 | 13987 |
| Slou.new.7 | 4 | B | TTAATTG | 1.53E-06 | 2.85E-05 | 18 | 1197 | 60 | 13987 |
| Rx.new.7 | 4 | B | CTAATTA | 6.16E-06 | 0.0001 | 17 | 1185 | 60 | 13987 |
| Odsh.new.7 | ? | B | CTAATTA | 6.37E-06 | 0.0001 | 17 | 1188 | 60 | 13987 |
| Pph13.new.7 | 4 | B | CTAATTA | 2.22E-05 | 0.0003 | 16 | 1168 | 60 | 13987 |
| Lim3.new.7 | 4 | B | TTAATTA | 2.29E-05 | 0.0003 | 16 | 1171 | 60 | 13987 |
| Hmx.new.7 | 4 | B | TTAATTG | 2.34E-05 | 0.0003 | 16 | 1173 | 60 | 13987 |
| Unpg.new.7 | 4 | B | TTAATTA | 2.80E-05 | 0.0003 | 16 | 1190 | 60 | 13987 |
| PdhP.new.7 | 4 | B | TTAATTA | 2.83E-05 | 0.0003 | 16 | 1191 | 60 | 13987 |
| Repo.new.7 | 4 | B | TTAATTA | 2.83E-05 | 0.0003 | 16 | 1191 | 60 | 13987 |
| Achi.new.7 | ? | B | TGACAG | 0.0001 | 0.0008 | 15 | 1148 | 60 | 13987 |
| C15.new.7 | 3 | B | TTAATTA | 0.0001 | 0.0008 | 15 | 1172 | 60 | 13987 |
| Dll.new.7 | 4 | B | CTAATTA | 0.0001 | 0.0008 | 15 | 1174 | 60 | 13987 |
| Dr.new.7 | 4 | B | CCAATTA | 0.0001 | 0.0008 | 15 | 1174 | 60 | 13987 |
| Ubx.txt | 4 | F | CAATTA | 0.0001 | 0.0008 | 15 | 1177 | 60 | 13987 |
| CG4136.new.7 | 2 | B | TTAATTA | 0.0001 | 0.0009 | 15 | 1185 | 60 | 13987 |
| Bsh.new.7 | 4 | B | TTAATTG | 0.0001 | 0.0009 | 15 | 1191 | 60 | 13987 |
| H20.new.7 | 3 | B | TTAATTA | 0.0001 | 0.0009 | 15 | 1197 | 60 | 13987 |
| CG33980.new.7 | 2 | B | TTAATTA | 0.0001 | 0.0009 | 15 | 1199 | 60 | 13987 |
| CG11085.new.7 | ? | B | TTAATTG | 0.0001 | 0.0009 | 15 | 1204 | 60 | 13987 |
| ftz.txt | 4 | F | TAATTGNC | 0.0003 | 0.0023 | 14 | 1167 | 60 | 13987 |
| Otp.new.7 | 4 | B | TTAATTA | 0.0003 | 0.0023 | 14 | 1171 | 60 | 13987 |
| CG9876.new.7 | 2 | B | CTAATTA | 0.0004 | 0.0023 | 14 | 1177 | 60 | 13987 |
| CG340131.new.7 | ? | B | TTAATTG | 0.0004 | 0.0023 | 14 | 1182 | 60 | 13987 |
| CG15696.new.7 | 2 | B | TTAATTG | 0.0004 | 0.0024 | 14 | 1190 | 60 | 13987 |
| CG12361.new.7 | 4 | B | TTTATTA | 0.0004 | 0.0026 | 14 | 1201 | 60 | 13987 |
| CG7056.new.7 | 2 | B | TTAATTA | 0.0005 | 0.0029 | 14 | 1217 | 60 | 13987 |
| hkb.new.1 | ? | B | GGGGCGTGA | 0.0008 | 0.0046 | 12 | 986 | 60 | 13987 |
| Ind.new.7 | 4 | B | CTAATTA | 0.0011 | 0.0058 | 13 | 1165 | 60 | 13987 |
| Hgtx.new.7 | 4 | B | TTAATTA | 0.0012 | 0.0058 | 13 | 1175 | 60 | 13987 |
| Zen2.new.7 | 4 | B | TTAATTA | 0.0012 | 0.0058 | 13 | 1175 | 60 | 13987 |
| CG4328.new.7 | 3 | B | TTTATTG | 0.0012 | 0.0058 | 13 | 1177 | 60 | 13987 |
| E5.new.7 | 3 | B | TTAATTA | 0.0012 | 0.0058 | 13 | 1178 | 60 | 13987 |
| Ro.new.7 | 4 | B | CTAATTA | 0.0012 | 0.0058 | 13 | 1180 | 60 | 13987 |
| CG32105.new.7 | 4 | B | TTAATTA | 0.0013 | 0.0058 | 13 | 1184 | 60 | 13987 |
| Dfd.txt | 4 | F | TTAATTAT | 0.0013 | 0.0059 | 13 | 1190 | 60 | 13987 |
| Cad.new.7 | 4 | B | TTTATTA | 0.0015 | 0.0065 | 13 | 1204 | 60 | 13987 |
| BH1.new.7 | 4 | B | TTAATTG | 0.0015 | 0.0065 | 13 | 1208 | 60 | 13987 |
| ap.txt | 4 | F | TAATTA | 0.0018 | 0.0073 | 13 | 1226 | 60 | 13987 |
| Ems.new.7 | 3 | B | TTAATGA | 0.0031 | 0.0124 | 12 | 1147 | 60 | 13987 |
| Al.new.7 | 4 | B | CTAATTA | 0.0031 | 0.0126 | 12 | 1151 | 60 | 13987 |
| Exex.new.7 | 4 | B | CTAATTA | 0.0035 | 0.0135 | 12 | 1165 | 60 | 13987 |
| CG18599.new.7 | 4 | B | TTAATTA | 0.0035 | 0.0135 | 12 | 1167 | 60 | 13987 |
| zen.txt | 4 | F | CATTAAANTT | 0.0038 | 0.0141 | 12 | 1180 | 60 | 13987 |
| V_TATA_01 | 2 | T | GTATAAAAG | 0.0084 | 0.0295 | 11 | 1140 | 60 | 13987 |
| Hth.new.7 | 4 | B | TGACAG | 0.0095 | 0.0327 | 11 | 1159 | 60 | 13987 |
| Lab.new.7 | 4 | B | TTAATTA | 0.0105 | 0.0347 | 11 | 1176 | 60 | 13987 |
| sna.txt | ? | F | CCACTTGCT | 0.0105 | 0.0347 | 11 | 1176 | 60 | 13987 |
| Pb.new.7 | ? | B | TTAATTA | 0.0108 | 0.0350 | 11 | 1180 | 60 | 13987 |
| CG11294.new.7 | 4 | B | TTAATTA | 0.0115 | 0.0356 | 11 | 1191 | 60 | 13987 |
| Lbl.new.7 | 4 | B | TAATTA | 0.0116 | 0.0356 | 11 | 1192 | 60 | 13987 |
| prd.txt | 2 | F | CCATTAC | 0.0116 | 0.0356 | 11 | 1192 | 60 | 13987 |
| Btn.new.7 | 3 | B | TTAATGA | 0.0120 | 0.0364 | 11 | 1198 | 60 | 13987 |
| ttk.new.6 | ? | B | AAGGATAAT | 0.0136 | 0.0385 | 10 | 1055 | 60 | 13987 |
| I_SRYBETA_Q6 | ? | T | AGAGATGCG | 0.0203 | 0.0568 | 9 | 959 | 60 | 13987 |
| Vis.new.7 | ? | B | TGACAG | 0.0250 | 0.0691 | 10 | 1162 | 60 | 13987 |
| Eve.new.7 | 4 | B | CTAATGA | 0.0254 | 0.0694 | 10 | 1165 | 60 | 13987 |
| Lim1.new.7 | ? | B | TTAATTA | 0.0261 | 0.0702 | 10 | 1170 | 60 | 13987 |
| Scr.new.7 | 4 | B | TTAATGA | 0.0266 | 0.0702 | 10 | 1174 | 60 | 13987 |
| Awh.new.7 | 4 | B | TTAATTA | 0.0275 | 0.0706 | 10 | 1180 | 60 | 13987 |
| Antp.new.7 | 4 | B | TTAATGA | 0.0277 | 0.0706 | 10 | 1182 | 60 | 13987 |
| vvl.txt | ? | F | TATGCA | 0.0293 | 0.0730 | 10 | 1193 | 60 | 13987 |
| toy.txt | ? | F | CCCCTCACTCAT | 0.0341 | 0.0840 | 9 | 1051 | 60 | 13987 |
| slbo.txt | 4 | F | ATTGCAAA | 0.0392 | 0.0954 | 9 | 1078 | 60 | 13987 |
| kruppel | 2 | T | AAAAGGGTT | 0.0465 | 0.1121 | 9 | 1113 | 60 | 13987 |
| nubbin.new.1 | 4 | B | TATGCAAATGA | 0.0499 | 0.1190 | 9 | 1128 | 60 | 13987 |

Motif source: B, B1H; F, flyreg.org data; T, Transfac

^a^Motif conservation score

^b^Number of genes common in motif targets and genes in the gene set

^c^Number of motif target genes

^d^Number of genes in the gene set

^e^Total number of genes in the analysis

Table S7D. Enrichment p-values for gustatory receptor genes in *Drosophila*

| **Motif** | **MCS**^a^ | **Motif**  **source** | **Motif best site** | **p-value** | **q-value** | **#common^b^** | **#motif targets^c^** | **#genes in gene set^d^** | **#total^e^** |
| --- | --- | --- | --- | --- | --- | --- | --- | --- | --- |
| I_ABDB_01 | 4 | T | GCNTTTATGGC | 0.0014 | 0.3117 | 13 | 1194 | 60 | 13987 |
| Dstat | ? | T | TTCCGGAA | 0.0030 | 0.3409 | 11 | 995 | 60 | 13987 |
| I_SN_01 | ? | T | GACAGGTGCA | 0.0057 | 0.3882 | 11 | 1080 | 60 | 13987 |
| hkb.new.1 | ? | B | GGGGCGTGA | 0.0087 | 0.3882 | 10 | 986 | 60 | 13987 |
| zen.txt | 4 | F | CATTAAANTT | 0.0108 | 0.4031 | 11 | 1180 | 60 | 13987 |
| ttk.new.6 | ? | B | AAGGATAAT | 0.0136 | 0.4342 | 10 | 1055 | 60 | 13987 |
| kruppel | 2 | T | AAAAGGGTT | 0.0191 | 0.4450 | 10 | 1113 | 60 | 13987 |
| Hsf.txt | 2 | F | TCTAGAANTTTCGA | 0.0209 | 0.4450 | 9 | 964 | 60 | 13987 |
| Al.new.7 | 4 | B | CTAATTA | 0.0236 | 0.4450 | 10 | 1151 | 60 | 13987 |
| Ind.new.7 | 4 | B | CTAATTA | 0.0254 | 0.4450 | 10 | 1165 | 60 | 13987 |
| bicoid.new.5 | 2 | B | TTAATCT | 0.0288 | 0.4450 | 10 | 1189 | 60 | 13987 |
| I_CROC_01 | 4 | T | AANAATAAATAT | 0.0293 | 0.4450 | 10 | 1193 | 60 | 13987 |
| vvl.txt | ? | F | TATGCA | 0.0293 | 0.4450 | 10 | 1193 | 60 | 13987 |
| run.new.6 | 4 | B | TTGCGGTTA | 0.0486 | 0.5229 | 9 | 1122 | 60 | 13987 |

Motif source: B, B1H; F, flyreg.org data; T, Transfac

^a^Motif conservation score

^b^Number of genes common in motif targets and genes in the gene set

^c^Number of motif target genes

^d^Number of genes in the gene set

^e^Total number of genes in the analysis

Table S8. Enrichment p-values for chemoreceptor gene sets, shown here for motifs that are significantly associated (p-value < 0.05) in at least two of the four tests performed: two for the species – *Drosophila* (D.mel) and *Nasonia* (N.vit), and two for the type of sense – olfactory (OR) and gustatory (GR).

| **Motif** | **MCS**^a^ | **Motif**  **source** | **Motif best site** | **N.vit**  **OR^b^** | **N.vit**  **GR^b^** | **D.mel**  **OR^b^** | **D.mel**  **GR^b^** |
| --- | --- | --- | --- | --- | --- | --- | --- |
| ttk.new.6 | ? | B | AAGGATAAT | 0.0128 | - | 0.0136 | - |
| kruppel.new.2 | 2 | B | CNAAAGGGTTA | 0.0013 | - | - | 0.0380 |
| run.new.6 | 4 | B | TTGCGGTTA | 0.0025 | - | - | 0.0486 |
| I_SN_01 | ? | T | GACAGGTGCA | 0.0028 | 0.0018 | - | 0.0057 |
| PR1 | ? | L | CCAAT | 0.0033 | 0.0120 | - | - |
| Exd.new.7 | 4 | B | TGACAA | 0.0052 | 0.0048 | - | - |
| slbo | 4 | F | ATTGCAAA | - | 0.0139 | 0.0392 | - |
| Lbl.new.7 | 4 | B | TAATTA | - | 0.0232 | 0.0116 | - |
| Dfd | 4 | F | TTAATTAT | - | 0.0246 | 0.0013 | - |
| I_CROC_01 | 4 | T | AANAATAAATAT | - | 0.0442 | - | 0.0293 |

Motif source: B, B1H; F, flyreg.org data; T, Transfac; L, literature

^a^Motif conservation score

^b^Enrichment p-values

**References**

1. Robertson HM, Warr CG, Carlson JR (2003) Molecular evolution of the insect chemoreceptor gene superfamily in Drosophila melanogaster. Proc Natl Acad Sci U S A 100 Suppl 2: 14537-14542.

2. Robertson HM, Gadau J, Wanner KW (2010) The insect chemoreceptor superfamily of the parasitoid jewel wasp Nasonia vitripennis. Insect Molecular Biology (In Press).

3. Zhang D, Zhou W, Yin C, Chen W, Ozawa R, et al. (2006) Misexpression screen for genes altering the olfactory map in Drosophila. Genesis 44: 189-201.

4. Ray A, van Naters WG, Shiraiwa T, Carlson JR (2007) Mechanisms of odor receptor gene choice in Drosophila. Neuron 53: 353-369.
